# KHDC3L mutation causes recurrent pregnancy loss by inducing genomic instability of human early embryonic cells

**DOI:** 10.1371/journal.pbio.3000468

**Published:** 2019-10-14

**Authors:** Weidao Zhang, Zhongliang Chen, Dengfeng Zhang, Bo Zhao, Lu Liu, Zhengyuan Xie, Yonggang Yao, Ping Zheng

**Affiliations:** 1 State Key Laboratory of Genetic Resources and Evolution, Kunming Institute of Zoology, Chinese Academy of Sciences, Kunming, China; 2 Yunnan Key Laboratory of Animal Reproduction, Kunming Institute of Zoology, Chinese Academy of Sciences, Kunming, China; 3 Kunming College of Life Science, University of Chinese Academy of Sciences, Kunming, China; 4 Key Laboratory of Animal Models and Human Disease Mechanisms of the Chinese Academy of Sciences & Yunnan Province, Kunming Institute of Zoology, Kunming, China; 5 Department of Obstetrics and Gynaecology, Yan An Hospital, Kunming Medical University, Kunming, China; 6 Yunnan Key Laboratory for Fertility Regulation and Birth Health of Minority Nationalities, Key Laboratory of Preconception Health in Western China, NHFPC, Population and Family Planning Institute of Yunnan Province, Kunming, China; 7 KIZ/CUHK Joint Laboratory of Bioresources and Molecular Research in Common Diseases, Kunming Institute of Zoology, Chinese Academy of Sciences, Kunming, China; 8 Center for Excellence in Animal Evolution and Genetics, Chinese Academy of Sciences, Kunming, China; The University of Texas at Austin, UNITED STATES

## Abstract

Recurrent pregnancy loss (RPL) is an important complication in reproductive health. About 50% of RPL cases are unexplained, and understanding the genetic basis is essential for its diagnosis and prognosis. Herein, we report causal KH domain containing 3 like (*KHDC3L*) mutations in RPL. *KHDC3L* is expressed in human epiblast cells and ensures their genome stability and viability. Mechanistically, KHDC3L binds to poly(ADP-ribose) polymerase 1 (PARP1) to stimulate its activity. In response to DNA damage, KHDC3L also localizes to DNA damage sites and facilitates homologous recombination (HR)-mediated DNA repair. KHDC3L dysfunction causes PARP1 inhibition and HR repair deficiency, which is synthetically lethal. Notably, we identified two critical residues, Thr145 and Thr156, whose phosphorylation by Ataxia-telangiectasia mutated (ATM) is essential for KHDC3L’s functions. Importantly, two deletions of KHDC3L (p.E150_V160del and p.E150_V172del) were detected in female RPL patients, both of which harbor a common loss of Thr156 and are impaired in PARP1 activation and HR repair. In summary, our study reveals both *KHDC3L* as a new RPL risk gene and its critical function in DNA damage repair pathways.

## Abstract 2

Recurrent pregnancy loss is an important complication in reproductive health, and about 50% of cases remain unexplained. This study shows that KHDC3L safeguards the genomic stability of human early embryonic cells, and damaging mutations in its gene cause recurrent pregnancy loss in humans.

## Introduction

Recurrent pregnancy loss (RPL) is defined as two or more spontaneous clinical pregnancy losses, according to the American Society for Reproductive Medicine [1]. It occurs in 1%–2% women attempting pregnancy, and the etiology is poorly understood [2,3]. The causes of pregnancy loss vary over the gestational stages. In the first trimester, more than half of the pregnancy loss is caused by chromosomal abnormalities of fetal tissues, including aneuploidy, polyploidy, and chromosome structural changes [3,4]. These chromosomal abnormalities are thought to arise mainly from meiotic errors, which lead to the generation of germ cells with aberrant chromosomes [3,5]. Aside from the meiosis-associated errors, other processes also contribute to the occurrence of chromosomal aberrations and early pregnancy loss. One such potential risk is the fast cell proliferation of early embryos. Frequent DNA replication and cell division are the major sources of endogenous DNA double-strand breaks (DSBs), which in turn cause variable chromosomal abnormalities [6]. Moreover, early embryonic cells have a unique cell-cycle profile and lack certain cell-cycle checkpoints [7]. Thus, the fast DNA synthesis in the early embryos is considered to be mutagenic. Meanwhile, early embryos at gastrulation stage are very sensitive to DSBs. The presence of a few DSBs in mouse gastrulating cells is sufficient to induce apoptosis and early embryo death [8]. Studies in mouse models showed that perturbations in molecular machineries ensuring the fidelity of DNA replication and cell division of early embryonic cells cause early pregnancy loss. For instance, depletion of factors such as Minichromosome maintenance protein 2–7 (Mcm2-7), Ataxia-telangiectasia and Rad3-related protein (Atr), breast cancer 2 (Brca2), RAS associated with diabetes protein 51 (Rad51), and pol(ADP-ribose) polymerase 1 and 2 (Parp1/Parp2) caused early embryonic lethality during gastrulation [9–16]. However, to the best of our knowledge, the association of human RPL with genomic stability–regulating genes in early embryos has not been explored.

Our previous works identified Khdc3 (also known as Filia and ES cell-associated transcript 1 [Ecat1]) as a key regulator in safeguarding the genomic stability of mouse early embryonic cells [17–19]. *Khdc3* is highly transcribed in oocytes and epiblast cells of pre- and postimplantation early embryos [20,21]. It is also expressed in mouse embryonic stem cells (ESCs) [22]. Depletion of Khdc3 protein from oocytes revealed its critical functions in ensuring euploidy and preventing micronuclei in cleavage-stage embryos [19]. Without maternal Khdc3, cleavage-stage embryos display high frequencies of aneuploidy and micronuclei formation, leading to preimplantation developmental arrest or delay and, consequently, reduced fecundity [19]. Using mouse ESCs as a model, we further revealed that zygotic Khdc3, which is expressed in epiblast cells between the blastocyst stage and embryonic day (E) 5.5 [21], also safeguards the genomic stability of epiblast cells in pre- and postimplantation embryos. Depletion of Khdc3 in epiblast cells causes embryo death and absorption after implantation [18]. Despite the presence of *Khdc3* orthologs in human and monkey (official symbol *KHDC3L*, also known as *ECAT1* and *C6orf221*), the protein sequences were poorly conserved between rodents and primates (approximately 35% similarity). In addition, the early embryogenesis is under divergent regulation between rodents and primates [23–26]. Human KHDC3L is expressed in oocytes [27,28] and was proposed to interact with NLR family pyrin domain containing 7 (NLRP7) to regulate the epigenetic status of the oocyte genome [29,30]. Maternal KHDC3L also interacts with three other maternal proteins to form a subcortical maternal complex, which persists in oocytes and preimplantation embryos [27]. Biallelic mutations of maternal KHDC3L cause familial biparental hydatidiform mole [31]. This phenotype is distinct from that observed in mice [19]. Although *KHDC3L* mRNAs were rarely detected in human and monkey morulae, the transcripts’ level increased dramatically in blastocysts [23–25,32]. Moreover, *KHDC3L* mRNAs were predominantly detected in epiblast cells by single-cell RNA sequencing in monkey embryos [23,25], and its expression level remained high until E14 at the onset of gastrulation [23]. These expression patterns suggest that KHDC3L may play a role during postimplantation embryogenesis. Accordingly, the current study aimed to understand the role of KHDC3L in human postimplantation embryos as well as its possible involvement in etiology of RPL. Because human ESCs (hESCs) are developmentally similar to the postimplantation epiblast cells [23,26], we utilized hESCs instead of human embryos for all the functional studies. Our results showed that *KHDC3L* mutations caused RPL by inducing severe genomic instability and apoptosis of early embryonic cells. We also revealed the divergent molecular mechanisms of human KHDC3L compared to mouse Filia.

## Results

### KHDC3L safeguards the genomic stability of hESCs

**Fig 1:**
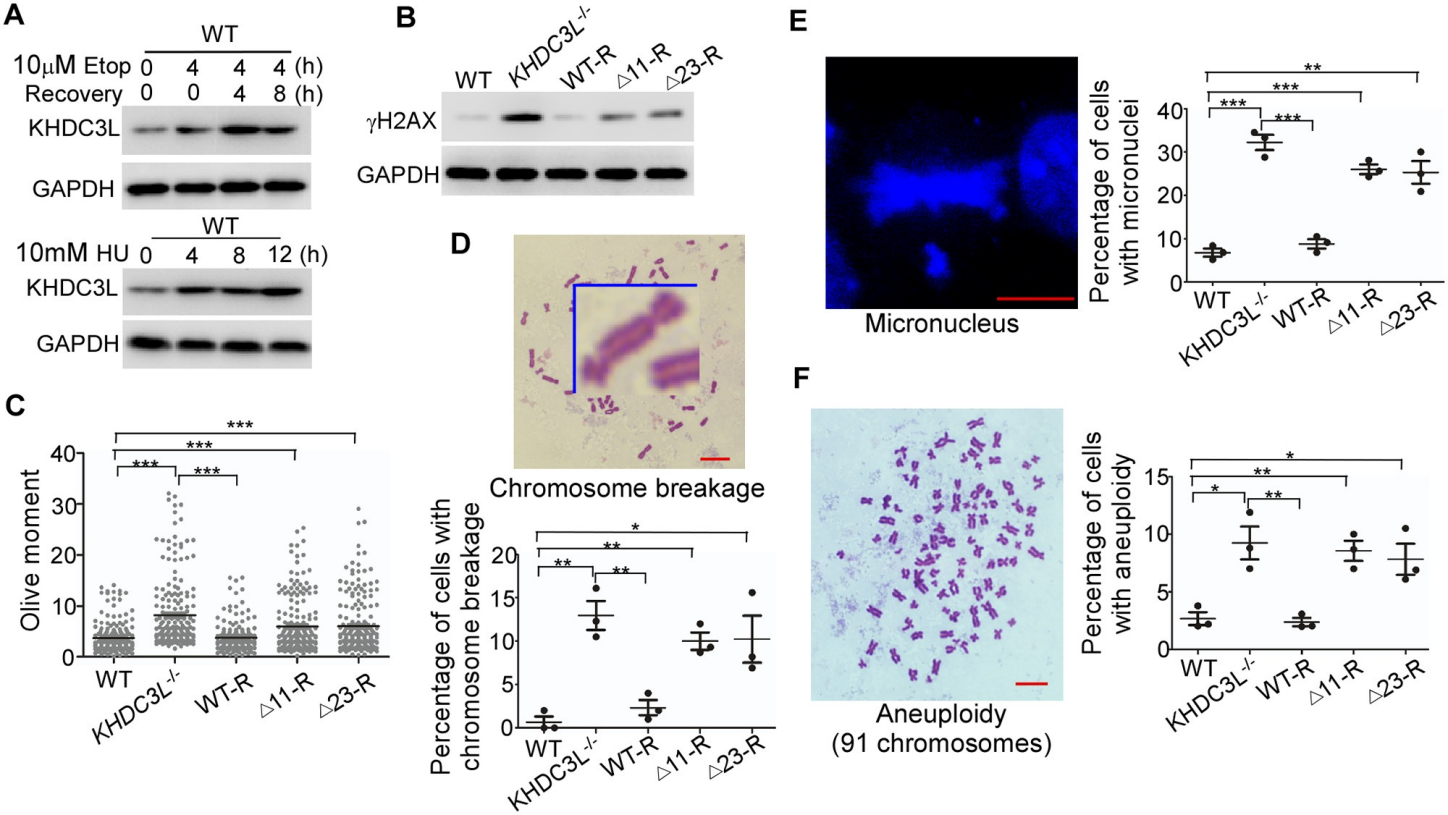
*KHDC3L* preserves genomic stability of hESCs. (A) KHDC3L protein expression was up-regulated by the treatments of Etop (upper panel) and HU (lower panel) in hESCs. Immunoblotting with γH2AX (B) and neutral comet assay (C) (*n* = 200 from two independent replicates) revealed that knockout of KHDC3L (*KHDC3L*^−/−^) in hESCs led to elevated DNA DSBs. This defect was fully rescued by reexpression of WT KHDC3L (WT-R) but not mutant proteins Δ11 or Δ23 (Δ11-R, Δ23-R) identified in patients with RPL. hESCs with dysfunctional KHDC3L (*KHDC3L*^−/−^, Δ11-R, Δ23-R) displayed a higher level of chromosome breaks (D), micronuclei (E), and aneuploidy (F) than those with functional KHDC3L (WT and WT-R) (*n* = 50 in one replicate, and total three independent replicates in D-F). Data represent mean ± SEM. *p < 0.05, **p < 0.01, ***p < 0.001. Scale bars, 10 μm. Underlying numerical values in (C-F) can be found in S1 Data. Δ11, p.E150_V160del; Δ23, p.E150_V172del; DSB, double-strand break; Etop, etoposide; GAPDH, glyceraldehyde 3-phosphate dehydrogenase; hESC, human embryonic stem cell; HU, hydroxyurea; KHDC3L, KH domain containing 3 like; WT, wild type.

*KHDC3L* is highly expressed in epiblast cells of human and monkey early embryos [23,25,32] (S1A Fig). To investigate the potential roles of KHDC3L in human early embryo development, we used hESCs as a surrogate for epiblast cells, which share similar molecular and developmental properties with ESCs [33]. Mouse Khdc3 regulates genomic integrity of epiblast cells and ESCs [17]; therefore, we tested whether human KHDC3L displays a similar function. Treatment of hESCs with DNA-damaging agent hydroxyurea or etoposide, which induces DNA replication stress and DNA DSBs, respectively [34], increased KHDC3L protein expression (Fig 1A). This suggested a potential involvement of KHDC3L in DNA damage response. We then knocked out *KHDC3L* at both alleles in hESCs using a CRISPR/Cas9-mediated strategy to investigate the function of KHDC3L in these cells (S1B Fig). DNA sequencing of the *KHDC3L* gene (S1C Fig) and immunoblotting analysis (S1D Fig) validated the complete deletion of KHDC3L in *KHDC3L-*knockout (*KHDC3L*^−/−^) hESCs. Wild-type (WT) KHDC3L proteins were then tagged with 3×Flag at the N terminus and expressed in *KHDC3L*^−/−^ ESCs to establish stably rescued ESCs (WT-R, S1E Fig). Knockout of *KHDC3L* in hESCs did not cause changes in morphology (S2A Fig), pluripotency marker expression (S2B Fig), proliferation rate (S2C Fig), cell-cycle distribution (S2D–S2F Fig), or long-term survival under the normal culture conditions.

However, examinations of the genomic integrity under the normal culture conditions by immunoblotting with γH2AX (a marker of DNA DSBs) [35] or neutral comet assay [36] revealed that *KHDC3L*^−/−^ hESCs displayed a high level of DNA DSBs. This defect was rescued by reexpression of WT KHDC3L proteins (Fig 1B and 1C). Concordantly, *KHDC3L*^−/−^ ESCs displayed higher rates of chromosome breakages (Fig 1D), micronuclei formation (Fig 1E), and aneuploidy (Fig 1F) compared to WT ESCs and WT KHDC3L-rescued ESCs. These results suggest that KHDC3L preserves the genome stability of hESCs and epiblast cells in early embryos. Its dysfunction may cause genomic instability in human early embryos, leading to the pregnancy loss at early gestational stages.

### Patients with RPL have deletions in *KHDC3L* that cause genomic instability

**Fig 2:**
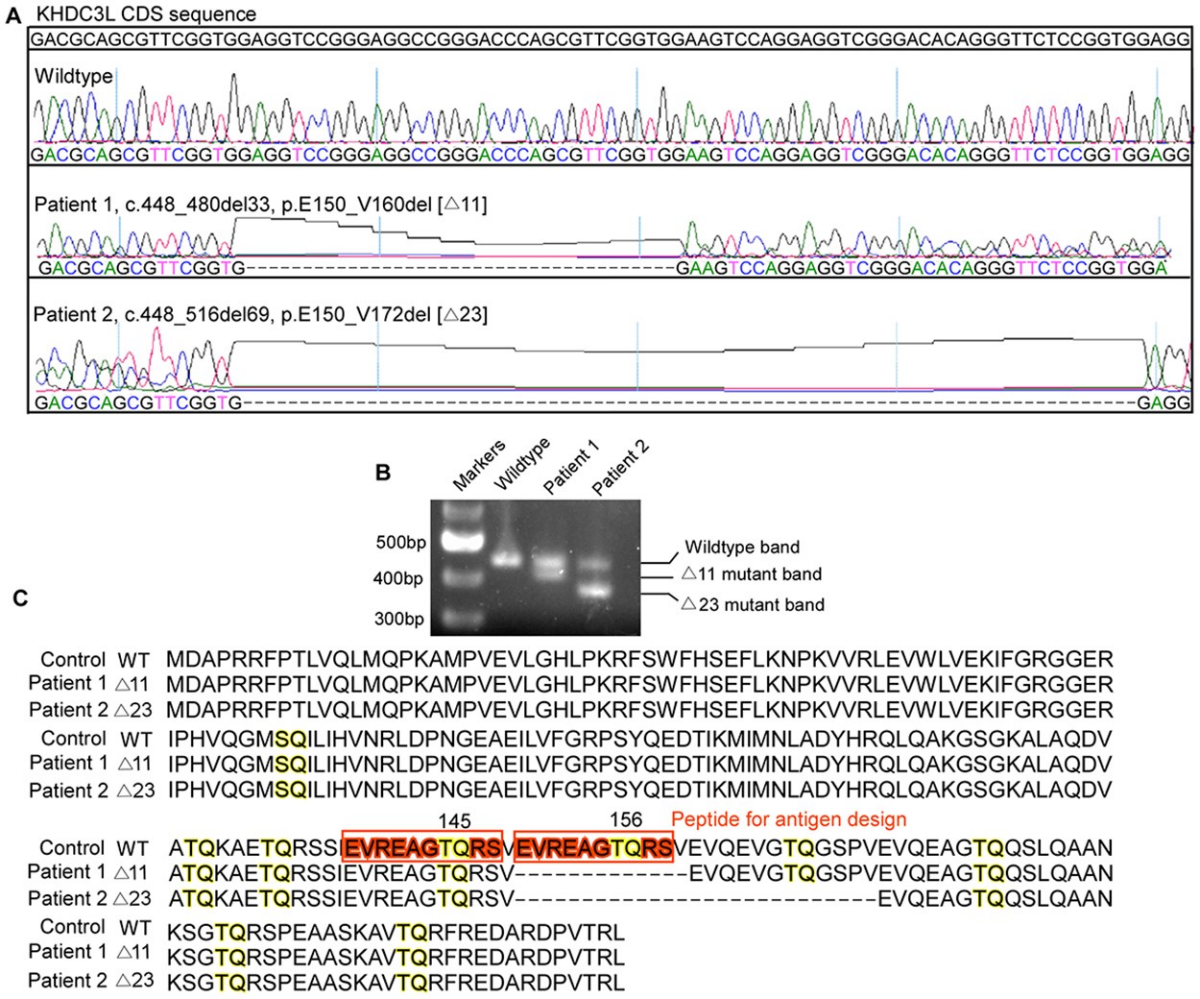
*KHDC3L* is mutated in patients with RPL. (A) Sanger sequencing identified Δ11 (NM_001017361, c.448-480del33) and Δ23 (NM_001017361, c.448_516del69) heterozygous deletions of *KHDC3L* in two unrelated patients. (B) PCR amplification confirmed the heterozygous mutations of *KHDC3L* in two patients. (C) Alignment of protein sequences of WT KHDC3L and two mutants (Δ11 and Δ23) identified in two unrelated patients with RPL. Δ11, p.E150_V160del; Δ23, p.E150_V172del; KHDC3L, KH domain containing 3 like; RPL, recurrent pregnancy loss; WT, wild type.

To investigate whether genomic mutations leading to *KHDC3L* deficiency are associated with RPL, we selected 29 female patients suffering from unexplained RPL. These patients undertook the common examinations for miscarriage-related factors, and etiologies including uterine factors, infection, and endocrine factors were excluded. We screened the entire *KHDC3L* gene in these patients and identified two heterozygous deletions of *KHDC3L* in two genetically unrelated patients (Fig 2A and 2B). Patient 1 had three pregnancy losses. She harbors a deletion (NM_001017361, c.448-480del33) encompassing 11 amino acids (aa) (p.E150_V160del, Δ11 for short). Patient 2 also had three pregnancy losses during the first trimester and had a deletion (NM_001017361, c.448_516del69) containing 23 aa (p.E150_V172del, Δ23 for short) (Fig 2C). Intriguingly, the 11-aa fragment overlaps with the 23-aa fragment (Fig 2C). We found no deletion in *KHDC3L* in 205 females with normal fertility, indicating that the deletion is significantly enriched in patients (2/29 versus 0/205, Fisher exact test, two-tailed *P* value = 0.015). Note that in the reference population from the ExAC database that contains 59,785 individuals [37], no such deletion was observed within the deleted region of *KHDC3L* (http://exac.broadinstitute.org/gene/ENSG00000203908). Compared with this reference population, the enrichment of *KHDC3L* deletion in RPL patients was even more significant (chi-squared test [Yates’ correction] *P* value < 2.2 × 10^−16^). In the Chinese Millionome Database (CMDB, https://db.cngb.org/cmdb/gene/1bc28f06dc9189dd4ac050b5f1866baf) [38], which contains whole-genome sequencing data generated for noninvasive prenatal testing from 141,431 Chinese women, there were also no such alterations. These data suggest that these deletions might be the causal variants for the unexplained RPL, albeit the patient sample size was relatively small.

We further investigated the potential functional significance of these patient-derived mutations. We expressed the mutant KHDC3L proteins (Δ11 and Δ23) tagged with 3×Flag in *KHDC3L*^−/−^ hESCs and established stable cell lines expressing the respective mutant (Δ11-R and Δ23-R, respectively, S1E Fig). Similar to the knockout, the deletion mutations of KHDC3L did not affect ESC morphology (S2A Fig), pluripotency marker expression (S2B Fig), proliferation rate (S2C Fig), cell-cycle distribution (S2D–S2F Fig), or long-term growth under the normal culture conditions. However, expression of the mutant proteins failed to rescue the defects of DNA DSBs and chromosomal abnormalities in *KHDC3L*^−/−^ cells (Fig 1B–1F), suggesting that these mutations led to loss of function and caused a wide range of genomic abnormalities in ESCs/epiblast cells.

### Dysfunction of KHDC3L may cause extensive genomic instability and cell death of early embryonic cells

**Fig 3:**
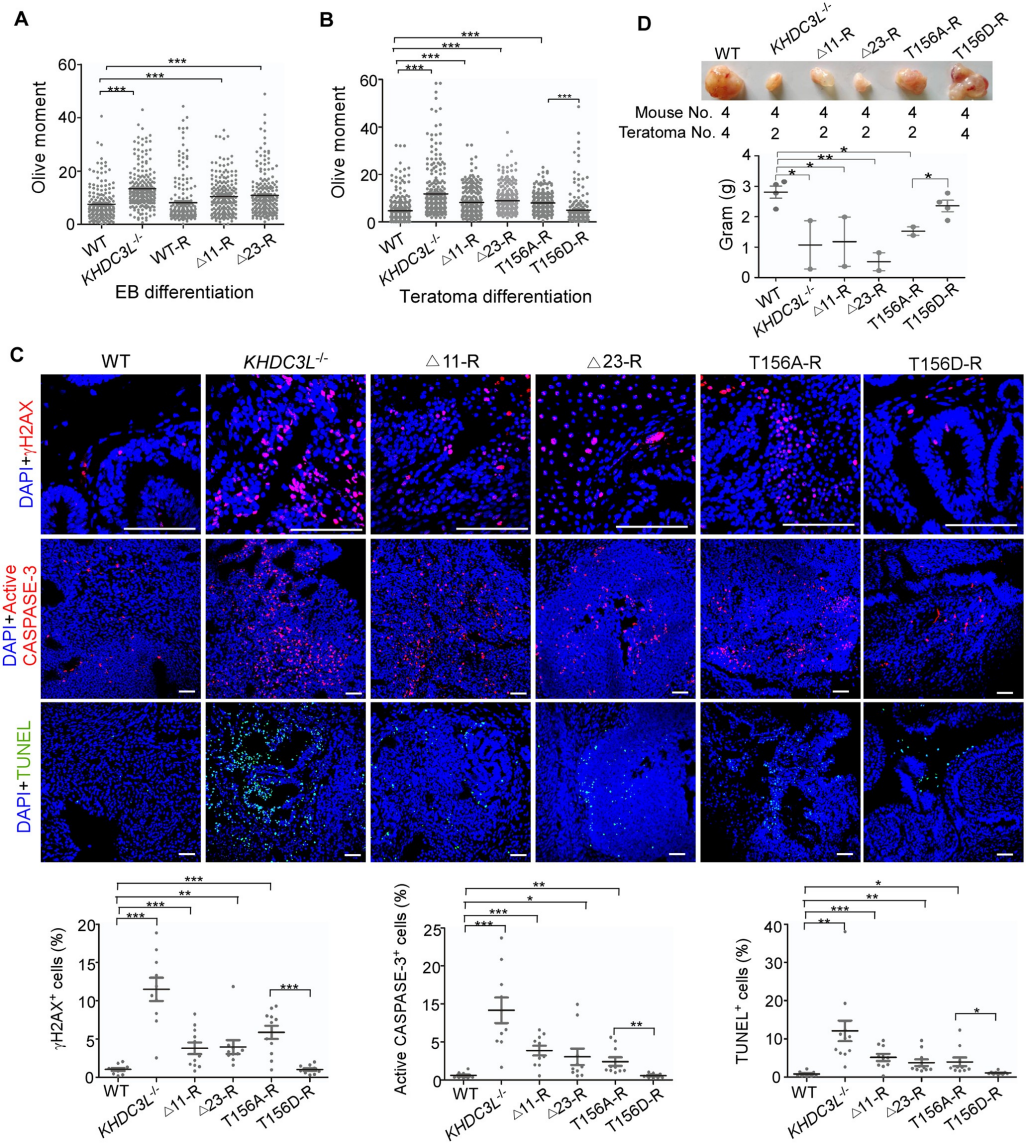
KHDC3L deficiency causes DNA damage and apoptosis in cells differentiated from hESCs. hESCs underwent in vitro EB differentiation (A) or in vivo teratoma differentiation (B). Neutral comet assay showed that differentiated progenies from hESCs with deficient KHDC3L (*KHDC3L*^−/−^, Δ11-R, and Δ23-R) had severe DNA DSBs (*n* = 200 from two independent experiments). (C) Immunostaining with γH2AX, active CASPASE-3, and TUNEL validated the higher level of DNA DSBs and apoptosis in teratoma cells differentiated from hESCs without KHDC3L or expressing the mutant KHDC3L (*KHDC3L*^−/−^, Δ11-R, Δ23-R, and T156A-R). (D) Fewer and smaller teratomas were formed by hESCs in the absence of functional KHDC3L (*KHDC3L*^−/−^, Δ11-R, Δ23-R, and T156A-R) (above, the injected mice numbers and the teratomas numbers; below, quantification of teratomas weight). Student two-tailed *t* test was performed for statistical analysis. Data represent mean ± SEM. *p < 0.05, **p < 0.01, ***p < 0.001. Scale bars, 100 μm. Underlying numerical values in A-D can be found in S1 Data. Δ11, p.E150_V160del; Δ23, p.E150_V172del; DSB, double-strand break; EB, embryoid body; TUNEL, TdT-mediated dUTP Nick-End Labeling; hESC, human embryonic stem cell; KHDC3L, KH domain containing 3 like; WT, wild type.

Epiblast cells are the founders of the embryogenesis. The genomic abnormalities of epiblast cells can be transmitted to their differentiated progenies. Based on the defects observed in *KHDC3L*-knockout or mutant hESCs, we hypothesized that KHDC3L dysfunctions may cause extensive genome aberrations in early embryonic cells. To this end, we performed in vitro differentiation of hESCs via the aggregation of embryoid bodies (EBs), in which ESCs undergo spontaneous differentiation into three germ layers [39]. The successful differentiation was confirmed by the diminished expression of pluripotency markers Nanog homeobox (*NANOG*) and POU class 5 homeobox 1 (*POU5F1*) (S3 Fig). Differentiated cells in EBs at day 15 were dissociated, and the genomic integrity was assessed by neutral comet assay. The progenies differentiated from hESCs without KHDC3L or with mutant KHDC3L displayed greater extent of DNA DSBs than those observed in hESCs containing WT KHDC3L (Fig 3A). To further validate the results, we injected hESCs into immunodeficient nonobese diabetic/severe combined immunodeficiency (NOD/SCID) mice to enable the in vivo differentiation into teratomas. At 56 d post differentiation, teratomas were harvested and examined. Consistently, neutral comet assay (Fig 3B) and immunostaining with γH2AX (Fig 3C) revealed that teratoma cells derived from hESCs with deficient KHDC3L (knockout, Δ11, or Δ23) contained a higher level of DNA DSBs than those derived from WT hESCs.

Genomic abnormalities impair cell viability and functions [40]. The extensive genomic abnormalities caused by KHDC3L deficiency in early embryonic cells suggested that KHDC3L dysfunction may induce early embryonic cell death. To test this hypothesis, we examined the apoptosis of in vivo differentiated cells from teratomas. Compared to the teratoma cells derived from WT hESCs, those from hESCs without KHDC3L or with mutant KHDC3L showed a higher rate of apoptosis, as indicated by the elevated positive staining for active CASPASE-3 and TdT-mediated dUTP Nick-End Labeling (TUNEL) (Fig 3C). Concordantly, fewer teratomas were formed in the absence of normal KHDC3L (Fig 3D). Moreover, teratomas derived from KHDC3L-knockout or mutant hESCs weighed less than those from hESCs containing functional KHDC3L (Fig 3D). Taken together, we conclude that KHDC3L safeguards the genomic stability and viability of early embryonic cells. The Δ11 and Δ23 mutations identified from RPL patients cause severe genomic abnormalities and apoptosis in early embryonic cells.

### KHDC3L does not participate in the replication stress response in safeguarding genomic stability of proliferating epiblast cells

We next sought to understand how KHDC3L deficiency leads to the genomic abnormalities of early embryonic cells. Similar to the ESCs, epiblast cells in early embryos proliferate fast and have a short G1 phase of the cell cycle [41], which renders them with high replication stress and at high risk to endogenous DNA damages [42]. We previously showed that mouse Khdc3 localizes on replication forks of ESCs, where it regulates the replication stress response to prevent endogenous DNA DSBs and chromosomal abnormalities [18]. We thus examined whether human KHDC3L ensures the genomic stability of epiblast cells through a similar pathway. hESCs were treated with hydroxyurea to induce replication-fork stalling; the fork restart ability was then measured by DNA fiber assay at different time points after hydroxyurea removal [18,43]. Neither the stalled fork restart nor the nascent DNA stability was affected by the absence or mutation of KHDC3L (S4A and S4B Fig). Concordantly, the ATR-checkpoint kinase 1 (CHK1) signaling, which coordinates the DNA replication stress response [44], was unaffected (S4C Fig). In line with these observations, KHDC3L did not localize on replication forks under the normal or hydroxyurea treatment condition (S4D Fig). Thus, unlike the mouse Khdc3, human KHDC3L does not participate in the regulation of replication stress response. Alternative mechanisms may underlie its role in regulating the genomic stability of human epiblast cells.

### KHDC3L independently regulates homologous recombination–mediated DNA repair and PARP1 activation, which are severely impaired by Δ11 and Δ23 mutations

**Fig 4:**
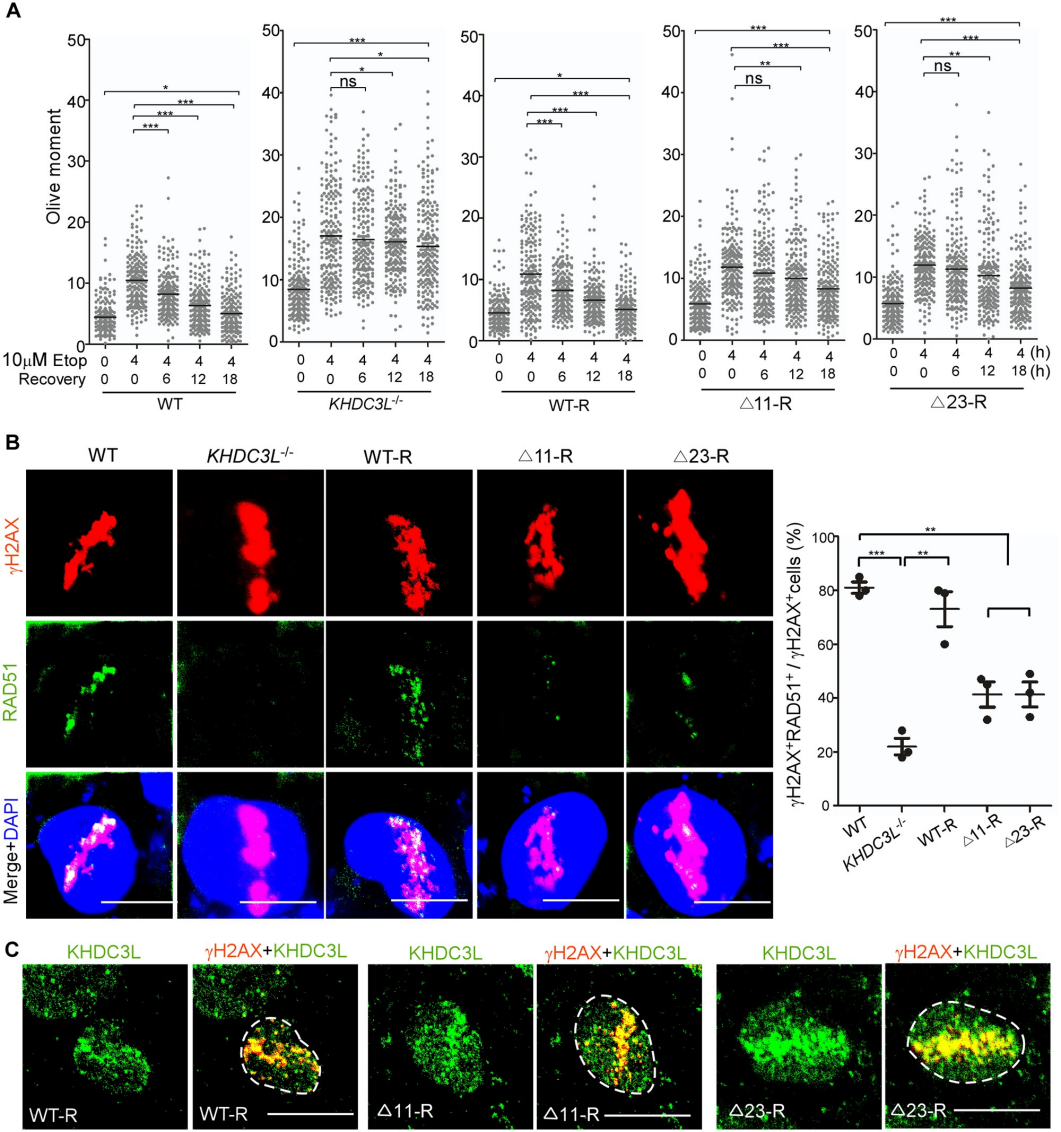
KHDC3L deficiency impairs HR-mediated DNA repair in hESCs. (A) Neutral comet assay revealed that DSB repair was less efficient in hESCs with deficient KHDC3L (*KHDC3L*^−/−^, Δ11-R, and Δ23-R) than in hESCs with proficient KHDC3L (WT and WT-R) (*n* = 200 from two independent experiments). (B) hESCs were laser micro-irradiated and examined following 2 h of recovery. hESCs with deficient KHDC3L (*KHDC3L*^−/−^, Δ11-R, and Δ23-R) are impaired in the recruitment of RAD51 to DSB sites labeled with γH2AX, indicating their reduced HR repair capacity (*n* = 50 in one replicate, total three independent replicates). (C) WT KHDC3L (WT-R) localizes to DSB sites. The Δ11 (Δ11-R) and Δ23 mutations (Δ23-R) did not impair this cellular localization (*n* = 10 in one replicate, total three independent replicates). Student two-tailed *t* test was performed for statistical analysis. Data represent mean ± SEM. *p < 0.05, **p < 0.01, ***p < 0.001. Scale bars, 10 μm. Underlying numerical values in (A) and (B) can be found in S1 Data. Δ11, p.E150_V160del; Δ23, p.E150_V172del; DSB, double-strand break; Etop, etoposide; hESC, human embryonic stem cell; HR, homologous recombination; KHDC3L, KH domain containing 3 like; ns, not significant; RAD51, RAS associated with diabetes protein 51; WT, wild type.

We then examined whether KHDC3L participated in the replication-associated DNA DSB repair to safeguard the genomic stability of epiblast cells. hESCs were treated with etoposide to induce DNA DSBs. The damage repair efficiency was evaluated by neutral comet assay following different time periods of recovery. Compared to WT hESCs, knocking out KHDC3L significantly decreased the DNA DSB repair efficiency. WT KHDC3L, but not Δ11 or Δ23 mutant, significantly rescued this defect (Fig 4A). To validate this result, we performed laser micro-irradiation to induce DNA DSBs in hESCs and examined the kinetics of focal γH2AX foci clearance. Consistently, focal γH2AX clearance was compromised in *KHDC3L*^−/−^ or Δ11-rescued ESCs (S5A Fig). Thus, KHDC3L participates in DSB repair, and the two patient-identified mutations impair this function. Consequently, hESCs without functional KHDC3L were more sensitive to DNA DSB insult (S5B Fig).

Replication-associated DNA DSBs are generally repaired via homologous recombination (HR)-mediated pathway, which occurs at the S and G2 phases of cell cycle and requires the recruitment of key recombinase RAD51 to DSB sites [45]. To determine whether KHDC3L regulates HR repair pathway, we examined the focal accumulation of RAD51 at DSB sites labeled with γH2AX. In hESCs containing WT KHDC3L, RAD51 localizes to γH2AX-positive DSB sites in the majority (approximately 80%) of cells after laser micro-irradiation (Fig 4B), and the recruitment efficiency did not change during the recovery (S5C Fig). However, absence or mutations in KHDC3L severely and persistently decreased the recruitment of RAD51 to DSB sites (Fig 4B; S5C Fig). Because KHDC3L does not affect cell-cycle distribution (S2D–S2F Fig), the decrease in the γH2AX^+^RAD51^+^ cell population could not be explained by the alterations in the S and G2 phases of the cell cycle. Rather, it suggested a bona fide impairment of HR repair by KHDC3L dysfunction. Of note, we found that KHDC3L localized on DSB sites to facilitate HR repair, the Δ11 and Δ23 deletions had no influence on this subcellular distribution (Fig 4C).

**Fig 5:**
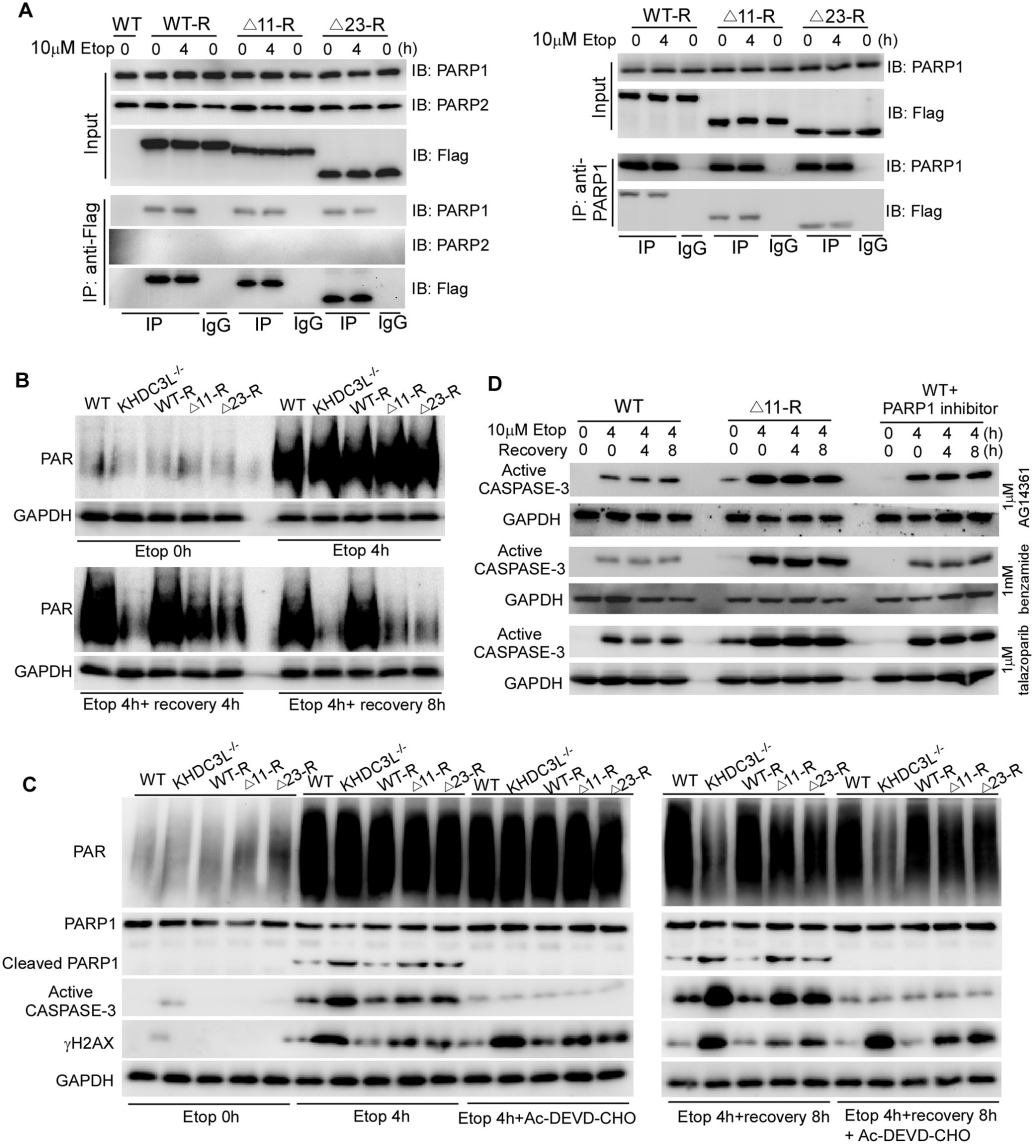
KHDC3L deficiency impairs PARP1 activation in hESCs. (A) Reciprocal coimmunoprecipitation revealed the physical and constitutive interaction of KHDC3L with PARP1 but not PARP2. The Δ11 or Δ23 mutation did not impair this interaction. (B) After Etop treatment, hESCs with proficient KHDC3L (WT and WT-R) maintained high PAR levels, whereas those with deficient KHDC3L (*KHDC3L*^−/−^, Δ11-R, and Δ23-R) failed to efficiently sustain PAR levels. (C) Apoptosis inhibitor Ac-DEVD-CHO successfully suppressed apoptosis and PARP1 cleavage. However, it did not affect the levels of PAR and γH2AX. (D) Due to synthetic lethality caused by simultaneous impairment of HR repair and PARP1 activation in hESCs with deficient KHDC3L, Δ11-R hESCs were more sensitive than WT hESCs or WT hESCs treated with the indicated PARP1 inhibitors in response to genotoxic insults. Δ11, p.E150_V160del; Δ23, p.E150_V172del; Etop, etoposide; GAPDH, glyceraldehyde 3-phosphate dehydrogenase; hESC, human embryonic stem cell; HR, homologous recombination; IB, immunoblot; IgG, immunoglobulin G; IP, immunoprecipitation; KHDC3L, KH domain containing 3 like; PAR, poly(ADP-ribose); PARP, PAR polymerase; WT, wild type.

PARPs catalyze the covalent attachment of the poly(ADP-ribose) (PAR) to proteins as a form of posttranslational modification (PARylation) and play important roles in DNA damage response [46]. PARP1 is a major PARP enzyme responsible for most of PAR production [17,46]. PARP1 is implicated in base excision repair, DNA single-strand break (SSB) repair, and nonhomologous end-joining (NHEJ)-mediated DSB repair [46] but not in HR-mediated DSB repair [47]. Inhibition of PARP1 can generate extensive DNA DSBs and lead to the enhancement of HR repair event [48]. Thus, simultaneous PARP inhibition and HR deficiency causes synthetic lethality [49,50]. We wondered whether human KHDC3L, as its mouse ortholog [17], simultaneously regulates the PARP1 activity, a synthetic lethal target of HR repair. Coimmunoprecipitation using antibody against Flag in hESCs reconstructed with 3×Flag-KHDC3L successfully pulled down PARP1. The association of KHDC3L with PARP1 was further validated by reciprocal immunoprecipitation (Fig 5A). Moreover, this interaction was not influenced by DNA damage or by two mutations (Fig 5A), indicating that KHDC3L physically and constitutively associated with PARP1. In contrast, no interaction was detected between KHDC3L and PARP2 (Fig 5A), another PARP family member responsible for PARylation [46]. We further investigated whether human KHDC3L modulated the enzymatic activity of PARP1 by monitoring the PAR level. The PAR level was significantly elevated and efficiently sustained in WT hESCs upon genotoxic treatment. However, it failed to be sustained after an initial rise in *KHDC3L*^−/−^ hESCs. This defect was fully rescued by WT KHDC3L but was only mildly restored by Δ11 or Δ23 mutants (Fig 5B).

We noticed that hESCs with deficient KHDC3L were prone to undergo apoptosis compared with those with WT KHDC3L (S5B Fig). Apoptosis can induce PARP1 cleavage (therefore its inactivation) and DNA fragmentation. To elucidate whether the defects in PARP1 activation, DNA breakages, and DNA repair observed in hESCs with deficient KHDC3L were caused by apoptosis, we treated the hESCs with two different CASPASE inhibitors, Ac-DEVD-CHO [51] and Z-DEVD fluoromethylketone (z-DEVD-fmk)[52,53], to block the apoptosis. CASPASE activation and PARP1 cleavage were successfully inhibited in hESCs (Fig 5C; S5D Fig). However, inhibitions of apoptosis and PARP1 cleavage had no influence on PARP1 activity (Fig 5C; S5D Fig), γH2AX level (Fig 5C; S5D Fig), or DNA DSBs repair by HR (S5E and S5F Fig). These observations support that KHDC3L regulates HR repair and PARP1 activation. In addition, during DNA damage response, there exist different phases of PARP1 activation, which are regulated differently. Specifically, KHDC3L is not required for the initial activation of PARP1 but is essential for sustaining PARP1 activity. Moreover, this function requires the fragments of KHDC3L that were deleted in RPL patients. Accordingly, we examined the ATM kinase activation, which is subject to PARP1 regulation [17,54]. ATM activation can be monitored by the phosphorylation of ATM at T1981 and of its downstream substrate CHK2 at T68 [55–57]. Upon DNA DSBs induced by 4-h etoposide treatment, ATM was activated and sustained in hESCs with proficient KHDC3L. However, its activity was impaired in hESCs with dysfunctional KHDC3L. Of note, the kinetics of ATM activity was similar to that of PARP1 activity in the absence of functional KHDC3L (S6A Fig). Taken together, these results support the notion that KHDC3L interacts with PARP1 and regulates its second wave of activation during DNA damage response. The deleted fragments of KHDC3L observed in RPL patients (Δ11 and Δ23) are not required for its interaction with PARP1 but are essential to regulate PARP1 activity.

To determine whether KHDC3L’s roles in HR repair rely on PARP1 activity in early embryonic cells, we inhibited PARP1 activity with PARP1 inhibitors including AG14361 [58], benzamide [59], and talazoparib (BMN-673) [60], which have distinct cytotoxic mechanisms (S6B–S6D Fig). Notably, blockage of PARP1 activity had no effects on RAD51 recruitment to DSB sites (S6B–S6D Fig), confirming that HR repair does not rely on PARP1 activity. Thus, KHDC3L regulates two independent events: HR-mediated DSB repair and PARP1 activation. KHDC3L dysfunction can simultaneously cause HR repair deficit and PARP1 inactivation, which leads to a synthetic lethal phenotype. Corroborating this conclusion, hESCs with dysfunctional KHDC3L (e.g., Δ11-R cells) were more sensitive to genotoxic insults than normal hESCs treated with PARP1 inhibitors (Fig 5D).

### The Δ11 and Δ23 mutations exhibit a dominant-negative effect

**Fig 6:**
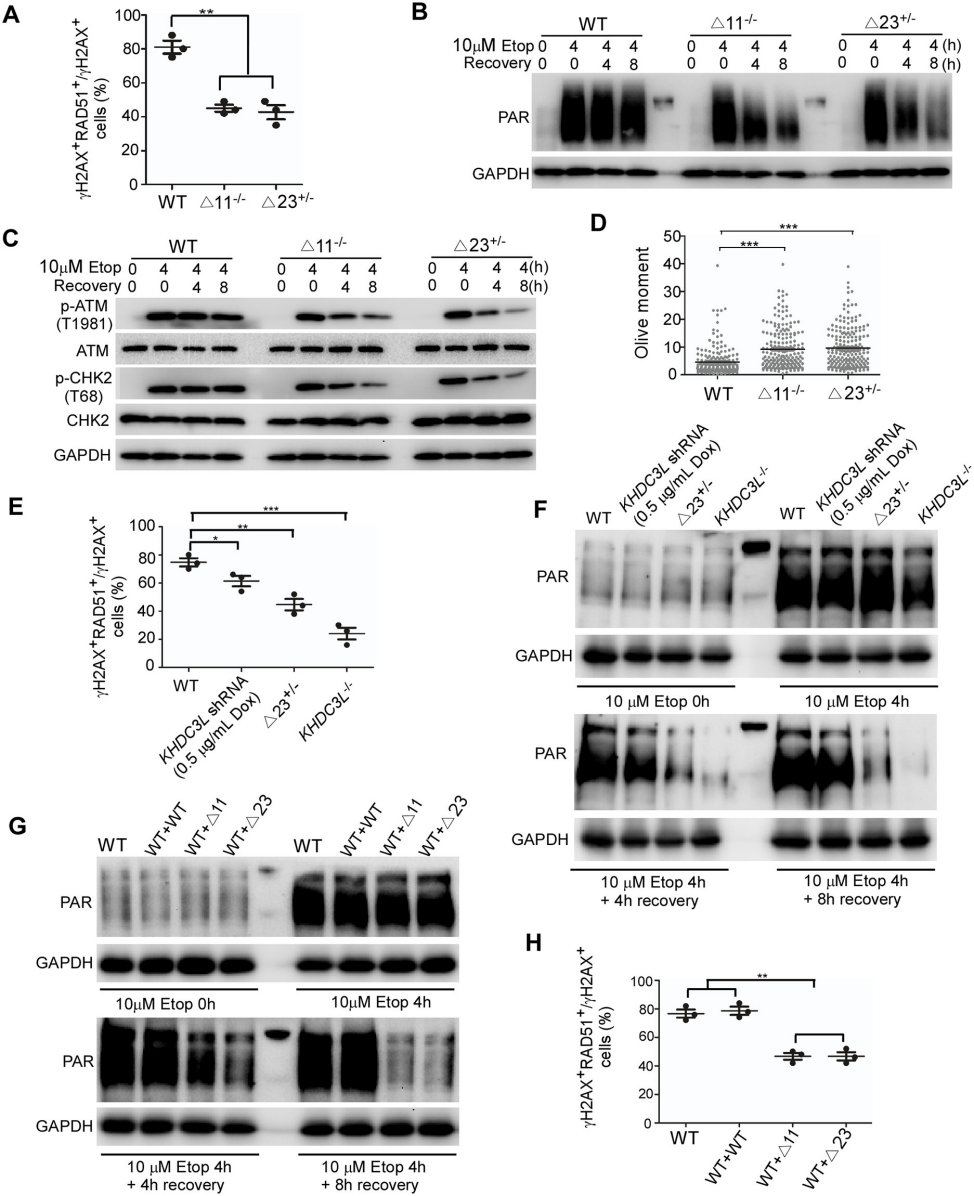
The Δ11 and Δ23 mutants display a dominant-negative effect. Homozygous deletion of 11aa (Δ11^−/−^) and heterozygous deletion of 23aa (Δ23^+/−^) in hESCs caused similar extent of defects in HR-mediated DNA repair (A) (*n* = 50 in one replicates, total three independent experiments), PARP1 activation (B), and ATM-CHK2 signaling (C). Consequently, Δ11^−/−^ and Δ23^+/−^ hESCs accumulated similar level of DNA DSBs (D) (*n* = 200 from two independent experiments). Compared to Δ23^+/−^ mutation, partial knockdown of KHDC3L caused milder defects in RAD51 recruitment to DSB sites (E) and PARP1 activation (F). Expression of Δ11 or Δ23 mutants, but not WT KHDC3L, in WT ESCs impaired PARP1 activation (G) and HR-mediated repair (H). Student two-tailed *t* test was performed for statistical analysis. Data represent mean ± SEM. *p < 0.05, **p < 0.01, ***p < 0.001. Underlying numerical values in (A), (D), (E), and (H) can be found in S1 Data. Δ11, p.E150_V160del; Δ23, p.E150_V172del; aa, amino acid; ATM, Ataxia-telangiectasia mutated; CHK2, checkpoint kinase 2; Dox, doxycycline; DSB, double-strand break; ESC, embryonic stem cell; Etop, etoposide; GAPDH, glyceraldehyde 3-phosphate dehydrogenase; hESC, human ESC; HR, homologous recombination; KHDC3L, KH domain containing 3 like; PAR, poly(ADP-ribose); PARP, PAR polymerase; shRNA, short hairpin RNA; WT, wild type.

The Δ11 and Δ23 mutant proteins’ localization to DNA DSB sites (Fig 4C) and interaction with PARP1 (Fig 5A) were not affected. However, they failed to efficiently regulate HR repair (Fig 4B) and PARP1 activation (Fig 5B). These observations suggest that the mutant proteins may display a dominant-negative effect by competing with WT KHDC3L. To test this hypothesis, we deleted the 11aa and 23aa from one or both alleles of *KHDC3L* gene by CRISPR/Cas9-mediated gene editing and compared their functional outcomes. We obtained the homozygous deletion of 11aa (Δ11^−/−^) and heterozygous deletion of 23aa (Δ23^+/−^) in hESCs (S7A and S7B Fig). Because the two mutations exhibited similar functional deficiency, we compared the abilities of HR repair and PARP1 activation among WT, Δ23^+/−^, and Δ11^−/−^ hESCs. Of note, Δ23^+/−^ and Δ11^−/−^ hESCs exhibited similar defects in HR repair (Fig 6A), PARP1 activation (Fig 6B), and ATM-CHK2 signaling (Fig 6C). Concordantly, they contained similar level of DNA DSBs (Fig 6D). These data suggest that Δ11 and Δ23 mutations may display a dominant-negative effect and that a single allele mutation is sufficient to penetrate phenotypically. We then utilized doxycycline-induced short hairpin RNA (shRNA) to partially knockdown KHDC3L and evaluate its effects. Compared to the Δ23^+/−^, approximately 50% depletion of KHDC3L (S7C Fig) caused milder decreases in HR repair efficiency (Fig 6E) and PARP1 activation (Fig 6F). Finally, we expressed WT and mutant KHDC3L (Δ11 and Δ23) in WT hESCs, respectively (S7D Fig). Compared with expression of WT KHDC3L in WT cells, expression of Δ11 or Δ23 mutant proteins in WT cells drastically impaired the PARP1 activity (Fig 6G) and HR repair (Fig 6H). Taken together, these lines of evidence support that Δ11 and Δ23 mutations exhibit a dominant-negative effect.

### Functional deficiency of Δ11 and Δ23 mutations is caused by the lack of T156 phosphorylation, which is required for HR repair and PARP1 activation

**Fig 7:**
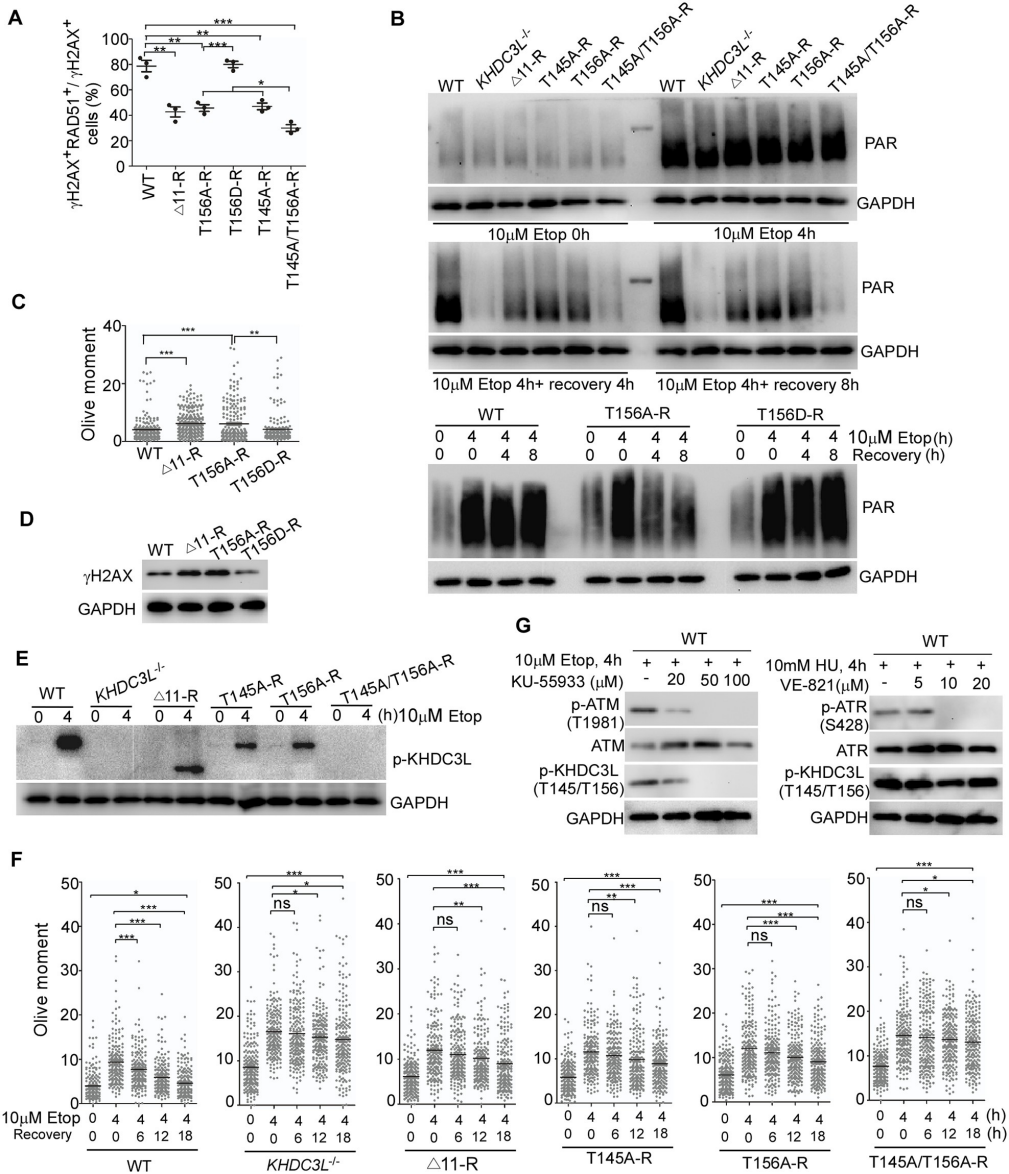
Phosphorylation of T156 and T145 sites in KHDC3L by ATM is required for HR repair and PARP1 activation. (A) T156A, T145A, and Δ11 mutations caused similar level of defect in recruiting RAD51 to DNA DSB sites, whereas T156D had no effects on RAD51 recruitment. Double mutations of T156A/T145A displayed a more severe phenotype than those of individual mutation (*n* = 50 in one replicate, total three independent experiments). (B) PARP1 activity in hESCs with WT or mutant KHDC3L proteins. Neutral comet assay (C) (*n* = 200 from two independent experiments) and immunoblotting (D) confirmed that hESCs expressing Δ11 or T156A mutant KHDC3L accumulated a higher level of DNA DSBs than did WT hESCs or ESCs expressing T156D. (E) Phosphorylation of T145/T156 sites at different hESC lines. (F) T145A, T156A, and Δ11 mutations displayed similar level of defects in DSB repair (*n* = 200 from two independent experiments). Similar to *KHDC3L*^−/−^, T156A/T145A double mutations caused more-severe defects. (G) Complete repression of ATM activation by KU-55933 eliminated the phosphorylation of T145/T156 in KHDC3L, whereas inhibition of ATR activity by VE-821 had no effects. Student two-tailed *t* test was performed for statistical analysis. Data represent mean ± SEM. *p < 0.05, **p < 0.01, ***p < 0.001. Underlying numerical values in (A), (C), and (F) can be found in S1 Data. Δ11, p.E150_V160del; ATM, Ataxia-telangiectasia mutated; ATR, Ataxia-telangiectasia and Rad3-related protein; DSB, double-strand break; ESC, embryonic stem cell; Etop, etoposide; GAPDH, glyceraldehyde 3-phosphate dehydrogenase; hESC, human ESC; HR, homologous recombination; KHDC3L, KH domain containing 3 like; ns, not significant; PAR, poly(ADP-ribose); PARP, PAR polymerase; RAD51, RAS associated with diabetes protein 51; WT, wild type.

We noticed that the C terminus of KHDC3L contains tandem TQ motifs (Fig 2C), which conform to the classical phosphorylation targets of ATM/ATR kinase [61]. Notably, the two deletions (Δ11 and Δ23) in RPL patients bear a common TQ motif (156TQ) (Fig 2C). This prompted us to test whether the common TQ motif is the phosphorylation site of ATM/ATR and the phosphorylation of T is critical to KHDC3L’s functions in regulating HR repair and PARP1 activation. To this end, we converted 156T to A (Ala as a nonphosphorylation mutation) or 156T to D (Asp as a constitutive phosphorylation mimics) and examined the functional significance of these conversions. Lentiviral vectors expressing T156A and T156D mutants were transfected into *KHDC3L*^−/−^ hESCs to establish stable cell lines (T156A-R and T156D-R ESCs, S8A Fig). Intriguingly, T156A-R hESCs displayed similar impairment in recruiting RAD51 to DSB sites (Fig 7A), sustaining a second wave of PARP1 activation (Fig 7B) and ATM-CHK2 signaling (S8B Fig) as in the Δ11-R cells. Consistently, T156A-R ESCs contained a level of endogenous DNA DSBs comparable to that of Δ11-R ESCs (Fig 7C and 7D). In contrast, T156D proteins functioned as efficiently as WT proteins and displayed no differences in RAD51 recruitment (Fig 7A), PARP1 activation (Fig 7B), ATM-CHK2 signaling (S8B Fig), or overall level of DNA DSBs compared with that of WT proteins (Fig 7C and 7D). Concordantly, teratomas differentiated from T156A-R ESCs were smaller, contained a high level of DNA damage, and underwent massive apoptosis, whereas those derived from T156D-R ESCs displayed no overt abnormalities (Fig 3B–3D). Taken together, these results support that T156 in KHDC3L is phosphorylated and that the phosphorylation is required for KHDC3L to regulate HR repair and PARP1 activation. Because T156A conversion phenocopied Δ11 and Δ23 mutations, we concluded that the functional deficiencies of these deletions were attributed to the loss of T156 phosphorylation. Notably, the mutations encompassing T156 were also reported in the ExAC database, which contains 59,785 individuals (p.Thr156Pro and p.Thr156AsnfsTer61, both occurred in only one individual) [37].

### Simultaneous phosphorylation of T156 and T145 by ATM is necessary for maintaining genomic stability of epiblast cells by KHDC3L

To further validate the phosphorylation of T156 and to identify its upstream regulatory kinase, we attempted to generate a polyclonal antibody for T156 phosphorylated human KHDC3L. In designing the antigen sequence, we found that the peptide sequence EVREAGTQRS was the most desirable. However, KHDC3L contains two EVREAGTQRS segments; the antibody would therefore recognize the phosphorylated KHDC3L at both T156 and T145 (Fig 2C). Using the antibody, we detected a specific single sharp band in WT but not in KHDC3L-knockout ESCs. The band intensity was significantly increased by etoposide treatment in WT cells but was less dramatically elevated in hESCs expressing T156A or Δ11 mutant proteins (Fig 7E). This result not only confirmed the phosphorylation of T156 but also implicated a potential modification of T145.

To verify the phosphorylation of T145 and to understand its functional consequence, we generated T145A mutation and T145A/T156A double mutations and established stable hESC lines expressing these mutant proteins (T145A-R, T145A/T156A-R, respectively, S8A Fig). Similar to T156A, T145A caused functional deficiency in RAD51 recruitment (Fig 7A), PARP1 activation (Fig 7B), ATM-CHK2 signaling (S8C Fig), and overall DNA DSB repair (Fig 7F). Notably, T145A/T156A double mutations caused more-severe phenotypes than did individual T156A or T145A mutations and phenocopied the KHDC3L knockout (Fig 7A, 7B and 7F; S8C Fig). Thus, T145 and T156 in KHDC3L are simultaneously phosphorylated and are required for KHDC3L’s function. Consistently, immunoblotting analysis revealed that the phosphorylation of KHDC3L at T145 and T156 was absent in hESCs expressing T145A/T156A double mutant proteins, whereas the phosphorylation level was reduced by approximately 50% in ESCs expressing individual T156A or T145A mutant when compared to WT ESCs (Fig 7E). TQ motif is the classical ATM/ATR phosphorylation target [61]. We then examined which kinase controlled the phosphorylation of T145 and T156 in KHDC3L. Blocking ATM activity by specific inhibitor KU-55933 [62] completely abolished T145 and T156 phosphorylation, whereas suppression of ATR activation via specific inhibitor VE-821 [63] had no influence (Fig 7G). Thus, ATM, but not ATR, catalyzes the phosphorylation of KHDC3L at T145 and T156.

In summary, we identified KHDC3L as a novel and unique substrate of ATM in epiblast cells of early human embryos, and the phosphorylation of KHDC3L plays a critical role in governing genomic integrity via two independent pathways: stimulating PARP1 activation and participating in HR-mediated DNA damage repair. Mutation of KHDC3L encompassing the phosphorylation sites T145 and/or T156 would compromise its dual functions and cause extensive genomic instability of early embryonic cells, thereby potentially leading to embryonic development failure and pregnancy loss.

## Discussion

Mouse early embryos at gastrulation stage are hypersensitive to DNA damage. The presence of a very low level of DNA DSBs in gastrulating embryonic but not extraembryonic cells could induce apoptosis and embryo death [8]. In addition, cytoplasmic micronuclei in embryonic cells can trigger the Cyclic GMP-AMP (cGAMP) synthase (cGAS)-synthase stimulator of interferon genes (STING) pathway to induce the inflammation, which interferes with pregnancy [10]. After embryo implantation, epiblast cells proliferate quickly, and DNA replication stress (as well as oxidative metabolites) represents a major source of intrinsic DNA damages [7]. Perturbations in DNA replication machinery—for instance, by depleting Atr [13], Brca2 [16], Rad51 [15], and Mcm2-7 in mice [10,14]—can cause replication-associated DNA DSBs, leading to early embryonic lethality. These mouse works highlight that preventing DNA DSBs and maintaining genomic stability of epiblast cells before the onset of gastrulation are important for successful embryo development. In monkey, KHDC3L is abundantly expressed in epiblast cells of early embryos until the gastrulation onset [23,25]. By utilizing hESCs as a surrogate of epiblast cells, we demonstrate that KHDC3L is critical in preventing the replication-associated DNA DSBs in epiblast cells. Importantly, by analyzing KHDC3L mutations identified in patients with RPL, our data identify *KHDC3L* as a risk gene for RPL. Moreover, we discovered two critical aa residues, Thr145 and Thr156, whose phosphorylation by ATM regulates KHDC3L’s function and whose mutations contribute to the etiology of RPL.

The implication of *KHDC3L* mutation in RPL was also reported in a Chinese patient who carried a recessive frameshift mutation and displayed a clinic symptom of partial hydatidiform moles plus RPL [64]. However, several other studies failed to find any *KHDC3L* mutation in a small cohort of patients (19, 15, 24, and 68 women, respectively) with recurrent miscarriages [65–68]. This discrepancy may either reflect that KHDC3L is not a common risk factor for RPL or simply be due to a small sample size and/or distinct genetic backgrounds of the patients. It is intriguing to understand why the patients harboring the deletion mutations of KHDC3L in our study could live to adulthood without severe health problems. This might be due to the fact that DNA damage incurred during early gastrulation perturbs normal embryonic development in an all-or-nothing fashion, as reported in mouse [8]. This phenomenon is observed in many other gene mutation cases, which show partial penetrance. Alternatively, these two KHDC3L mutations may arise during the early embryonic development, when the fast DNA synthesis is mutagenic [7,69,70]. Mutated KHDC3L was then transmitted to germline with no detectable phenotype, thus leading to the generation of a healthy organism at birth.

KHDC3L functions through two independent pathways: ensuring the HR-mediated DNA repair and stimulating PARP1 activation. Of note, regulations on both pathways require the ATM-catalyzed phosphorylation of Thr145 and Thr156, which synergistically control KHDC3L’s dual functions. Knocking out KHDC3L or simply losing one or two phosphorylation sites led to simultaneous HR suppression and PARP1 inactivation, which act in concert to cause synthetic lethality [49,50]. Indeed, robust DNA DSBs were detected in hESCs and in teratoma cells when KHDC3L was absent or mutated at Thr145 and/or Thr156 residues. Moreover, KHDC3L absence or mutations compromised the teratoma formation and decreased the teratomas size by triggering apoptosis. These results are consistent with the idea that KHDC3L dysfunction may cause human embryo developmental failure and pregnancy loss. Similarly, double depletion of Parp1 and Atm in mice causes early embryo lethality [9]. By screening the *KHDC3L* gene mutations in RPL patients, we indeed found two specific deletions of KHDC3L (Δ11 and Δ23) in two genetically unrelated patients suffering from three early pregnancy losses with unknown cause. These deletions were not detected in a cohort of control fertile women or in the ExAC database and CMDB [37,38]. The potential involvement of these mutations in RPL was further supported by a series of functional assays in hESCs. First, the mutant proteins in KHDC3L-knockout hESCs failed to rescue the extensive DNA damages and chromosomal aberrations in hESCs and their in vitro or in vivo differentiated progenies in teratomas. Differentiated cells in teratomas underwent robust apoptosis, leading to the reduced growth. Since both mutations detected in RPL patients are monoallelic, we made the Δ23 deletion in one allele and evaluated its functional outcomes. Our results showed that monoallelic mutation displayed an extent of deficiencies similar to that of biallelic mutation. Second, we uncovered the potential pathogenicity of the Δ11 and Δ23 deletions. These mutants did not impair KHDC3L recruitment to DSB sites or binding to PARP1, but failed to stimulate HR repair and PARP1 activation. Thus, by competing with WT proteins, these mutants display a dominant-negative effect. As a result, heterozygous mutation is sufficient to cause penetrating phenotype. Importantly, these two deletions bear a common loss of the critical phosphorylation site Thr156. This not only explained why the two mutations displayed similar extent of functional deficiencies but also highlighted the importance of these phosphorylation sites to the etiology of RPL. Other mutations encompassing Thr156 (e.g., p.Thr156Pro and p.Thr156AsnfsTer61) were also reported in the ExAC database [37] with a very low frequency. Although both Thr145 and Thr156 are of equal importance to the functions of KHDC3L, we did not find any mutation encompassing Thr145 in our patient cohort or in the ExAC database [37]. This suggests that Thr156 is more prevalent than Thr145 in RPL and that a larger patient cohort is needed to validate this hypothesis. Note that in the CMDB database, which contains whole-genome sequencing data generated for noninvasive prenatal testing from 141,431 Chinese women [38], we found no deletion or mutation encompassing Thr145 or Thr156. Since subjects in the CMDB underwent noninvasive prenatal testing and all the pregnancies were ongoing, it is reasonable to speculate that most CMDB subjects have normal fertility. Therefore, the absence of disruptive *KHDC3L* mutations in CMDB further supports a role of KHDC3L in RPL. Notably, the *KHDC3L* mutations in fetuses can be inherited maternally and/or paternally or de novo generated during early embryogenesis [69,70], and the frequency of damaging *KHDC3L* mutations responsible for RPL cases may be underestimated if the genetic screening is performed only in maternal genome.

Previous studies showed that *KHDC3L* mRNAs are also expressed in oocytes and early embryos prior to zygotic genome activation [23,25,27,28], and the protein persists during the preimplantation development [27,28]. This suggests that maternal KHDC3L may regulate oocyte growth, maturation, and early embryo development. In 2011, a pioneer study reported for the first time that biallelic *KHDC3L* mutations caused the familial complete hydatidiform mole [31], an extreme infertility condition that is characterized by early embryonic arrest and excessive trophoblastic proliferation. The observed phenotype was attributed to abnormal maternal gene imprinting [71,72]. Follow-up studies also identified several other biallelic mutations of *KHDC3L* that are causal to complete hydatidiform mole [68,73]. These studies proposed that maternal KHDC3L indirectly participates in the maternal gene imprinting during oogenesis or preimplantation embryogenesis by unknown mechanisms. Thus, maternal and zygotic KHDC3L proteins may function differentially and their deficiencies contribute to distinct types of developmental failure, i.e., familial complete hydatidiform mole and common RPL, respectively. Further studies are needed to solidify this speculation.

Although human KHDC3L and mouse Filia are ortholog, they seem to display divergent functions and molecular mechanisms. For instance, unlike in humans, loss of maternal Filia in mice does not cause complete hydatidiform mole [19]. Although both mouse Filia and human KHDC3L regulate genomic stability in epiblast cells [17,18], the underlying molecular pathways and mechanisms are divergent in the following aspects. First, mouse Filia can reside on stalled replication forks and regulate replication stress response [18], whereas human KHDC3L does not. Second, depletion of human KHDC3L induces robust impairment on HR-mediated DNA repair, whereas depletion of mouse Filia only mildly affects HR pathway [17]. Thus, by inhibition of PARP1 activation, the deficiency of human KHDC3L, but not mouse Filia, generates severe DNA damage and genomic instability via synthetic lethality between HR deficiency and PARP1 inhibition. Third, we uncovered human KHDC3L as a novel ATM kinase substrate, identified the phosphorylation sites, and elucidated the critical roles of phosphorylation in regulating KHDC3L’s functions. In contrast, whether mouse Filia is a substrate of Atm remains to be determined. Taken together, the present study provided critical insights into the role of human KHDC3L in regulating HR repair, PARP1 activation, and genomic stability and revealed human *KHDC3L* as a new RPL risk gene.

## Materials and methods

### Ethics statement

The mice care and use protocols adhered to the Guide for the Care and Use of Laboratory Animals and the Animal Welfare Act and were approved by the Institutional Animal Care and Use Committee of the Kunming Institute of Zoology (2015–012).

Human blood samples were collected from 29 females suffering from RPL and 205 females with normal fertility (defined as having at least one child and without pregnancy loss) from the Yan An Hospital affiliated to the Kunming Medical University. All participants gave their written, informed consent. This study was approved by the Institutional Review Board of Kunming Institute of Zoology, Chinese Academy of Sciences.

### hESC culture

hESCs (H9, a gift from Dr. Shaorong Gao) were cultured on mitomycin C–treated mouse embryonic fibroblasts (MEFs) in knockout serum replacement (KSR) medium containing 80% DMEM/F12 (DF12, Gibco, 12500–062), 20% KSR (Gibco, A31815-02), 2 mM L-Glutamine (Sigma, G8540-100G), 0.1 mM nonessential aa (NEAA, Gibco, 11140–050), 0.1 mM β-mercaptoethanol (Sigma, M3148-100ML), and 5 ng/mL bFGF (Millipore, GF003). Cultures were passaged every 3–4 d.

### *KHDC3L* gene editing in hESCs via CRISPR/Cas9

The PX330 plasmid was constructed and single-guide RNAs (sgRNAs) were designed as previously reported (http://crispr.mit.edu/) [74]. sgRNA AGGCGGTTTCCGACGCTCGT was designed to target exon 1 of *KHDC3L*. sgRNA was cloned into PX330. hESCs were dissociated into single cells using accutase (Gibco, A1110501) following 2 h preincubation with 10 μM Y-27632 (ROCK inhibitor, Selleck, S1049). Next, 5 μg Cas9 and guide RNA expression plasmids were transfected with 2 × 10^5^ hESCs by electroporation (Neon Transfection System, Life Technologies) according to the manufacturer’s instructions. Twenty-four hours after transfection, hESCs expressing red fluorescence protein were sorted by flow cytometry (Becton Dickinson, Influx cell sorter), and individual single cells were manually picked and transferred into one well of 96-well plates and maintained in mTeSR1 (Stem Cell Technology, 85850) supplemented with 10 μM Y-27632. Fifteen days after plating, individual colonies were picked up, and the *KHDC3L* gene sequence was determined by Sanger sequencing of PCR products. The following primers (forward primer: 5′-CTGCTCCTGACAGAAGGGAC and reverse primer: 5′-GCTCCAGGTAGCCCTATTCC) were used to amplify the genomic region flanking the CRISPR/Cas9 target site. ESC colonies with frameshift mutation of both *KHDC3L* alleles were expanded.

A pair of sgRNAs was used to precisely delete 11aa and 23aa, respectively, from KHDC3L. The sgRNAs used for deleting 11aa were 5′-CGCAGCGTTCGGTGGAGGTC-3′ (Δ11-sgRNA1) and 5′-CCAGCGTTCGGTGGAAGTCC-3′ (Δ11-sgRNA2). sgRNAs used for deleting 23aa were 5′-CTGGACTTCCACCGAACGCT-3′ (Δ23-sgRNA1) and 5′-GGCAGCCTGGAGAGACTGCT-3′ (Δ23-sgRNA2). The sgRNAs were cloned into PX330 plasmid. The PX330 plasmid and 5 μL single-stranded oligodeoxynucleotides (ssODNs) template (10 μM; Δ11^−/−^, AGCGGTCTTCAATAGAAGTCCGGGAGGCCGGGACGCAGCGTTCGGTGGAAGTCCAGGAGGTCGGGACACAGGGTTCTCCGGTGGAGGTGCAGGAGGCC; Δ23^+/−^, GGAGGCCGGGACGCAGCGTTCGGTGGAGGTCCGGGAGGCCGGGACCCAGCAGTCTCTCCAGGCTGCCAACAAGTCGGGGACCCAGCGATCCCCCGAAG) were cotransfected into hESCs as described above. hESCs were transfected as described above. The sequence of *KHDC3L* in individual ESC colonies was determined by Sanger sequencing of nested PCR products. For the nested PCR, the outer primer pair (forward primer: 5′-GATCCAGAAGGCCAAATTGAA-3′ and reverse primer: 5′-CATAATCTAGTAACTGGGTCC-3′) was used in the first round of PCR, and the inner primer pair (forward primer: 5′-CCCAGCGGTCTTCAATAGAA-3′ and reverse primer: 5′-GATGCCTTCATAATCTAGTAAC-3′) was used in the second round of PCR to amplify the genomic region flanking the CRISPR/Cas9 target site for the *KHDC3L* gene.

### Construction of lentiviral expression vectors

WT *KHDC3L* cDNA was amplified by PCR using TransStart FastPfu Fly DNA Polymerase (TRANSGEN BIOTECH, AP231-12) and cloned into pMD19-T Vector (Takara, 3271). pMD19-T vector containing WT *KHDC3L* was used as template to generate point mutation (T156A, T145A, T156D) by PCR. T145/156A double mutations were generated by PCR using T156A as template. The Δ11 and Δ23 mutations were generated by overlapping PCR amplifications. For the overlapping PCR, the Forward-1/Reverse-1 and Forward-2/Reverse-2 primers were used respectively in the first round PCR, and the products of the first round PCR were then overlapped using Forward-1/Reverse-2 primers. A 3×Flag tag was synthesized into pMD19-T vector by Tsingke Biological Technology, and WT, Δ11, and Δ23 were fused with a 3×Flag tag at the N terminus. All the new amplified cDNAs were cloned into pTOMO-IRES-EGFP lentiviral expression plasmid. Primers used in making constructs were listed in S1 Table.

pTOMO-IRES-EGFP plasmids were cotransfected into 293T cells with packaging plasmids (PMD2G and psPAX2) to package viruses using Lipofectamine 2000 (Life Technologies) according to the manufacturer’s instructions. Infected hESCs expressing GFP were purified by FACS (Becton Dickinson, Influx cell sorter).

### Karyotype analysis

hESCs maintained on Matrigel (Corning, 354277) at logarithmic phase were incubated with 120 ng/mL KARYOMAX Colcemid (Gibco, 15212–012) for 2 h at 37 °C in 5% CO_2_. hESCs were then treated with 0.05% trypsin-EDTA (Invitrogen, 25200072) at 37 °C for 2 min and centrifuged at 200*g* for 5 min. The cell pellets were gently resuspended in 0.075 M KCL and incubated for 20 min at 37 °C followed by fixation with methanol/glacial acetic acid (3:1). Fixed cells were dropped on microscope slides, air dried, and baked at 90 °C for 1 h. The slides were stained with 3% KaryoMAX Giemsa solution (Gibco, 10092013) at pH 6.8 for 10 min and examined by light microscopy. At least 50 metaphases were examined per cell line.

### Micronucleus assay

hESCs were cultured under normal conditions until approximately 70%–80% confluence. Cells were fixed with 4% (w/v) paraformaldehyde for 20 min on ice and subjected to DAPI staining. A total of 50 mitotic cells were analyzed per sample in one replicate. Three replicates total were performed.

### In vivo teratoma formation

Teratoma formation was performed according to the method described by Hannes Hentze [75] and our previous study [17]. In brief, NOD/SCID mice were purchased from Beijing Vital River Laboratory Animal Technology. Mice were anesthetized by intraperitoneal injection of 100 mg/kg pentobarbital (Sigma, P3761). About 1 million undifferentiated hESCs were subcutaneously injected into the hind leg muscle using an 18-gauge needle. At 8 wk, mice were euthanize and teratomas were collected and examined. Parts of teratoma tissues were fixed with PBS containing 4% paraformaldehyde and dehydrated in sucrose solution (10%, 20%, and 30%, respectively). The tissue sections (10 μm/section) were stained with TUNEL (Roche, 11684795910), active CASPASE-3, and γH2AX.

To examine the DNA damages of teratoma cells by comet assay, parts of teratoma tissues were dissected by finely mincing with a scalpel blade, followed by incubation with 2 mL of 2 mg/mL Collagenase IV (Gibco, 17104–019) for 10–15 min at 37 °C. The supernatants were then collected and 10 mL DMEM was added. The cell pellet was centrifuged and washed twice with DMEM and resuspended with DMEM for downstream use in comet assay.

### EB formation

EB was grown as previously described with slight modification [76]. Briefly, hESCs were detached from feeder cells by exposure to 1 mg/mL collagenase IV at 37 °C for 15 min. The detached colonies were then collected and transferred to agar-coated dishes and cultured in suspension in differentiation medium consisting of 80% DF12, 20% FBS, 2 mM L-Glutamine, 0.1 mM NEAA, 0.1 mM β-mercaptoethanol, 100 U/mL penicillin, and 100 μg/mL streptomycin. The medium was changed every 2–3 d. After 7 d of floating culture, the EBs were transferred to Matrigel-coated plates and cultured in the same medium for another 8 d for further differentiation. Medium was changed every other day.

### Quantitative real-time PCR analysis

For quantitative real-time PCR analysis of gene expression, total RNAs were extracted using RNeasy Plus Kit (Qiagen, DP419). Total RNA (1 μg) was reverse transcribed using PrimeScript RT reagent Kit (Perfect Real Time) (Takara, RR037A). The cDNAs were then amplified using SYBR green master mix (Takara, RR820B) on an ABI7300 (Applied Biosystems) thermo-cycler by specific primers. GAPDH was used as an internal control, and signals in each sample were normalized against it. Primers used for detection of *NANOG*, *POU5F1*, and *GAPDH* expression were listed in S1 Table.

### Antibodies information

Threonine 145/156 phospho-KHDC3L antibody was generated by Abmart. Synthetic peptide antigens (corresponding to EVREAGT[p]QRS) were utilized to immunize rabbit. All other primary and secondary antibodies were obtained commercially, and the information was listed in S2 Table.

### DNA fiber assay

The DNA fiber assay was performed as described in [77] and our previous study [18]. For stalled fork restart assay, cells were labeled with 25 μM 5-iodo-2′ -deoxyuridine (IdU, Sigma, I7125) for 30 min before washing three times with warm PBS. Cells were then treated with or without 10 mM hydroxyurea (Sigma, H8627) for 4 h. Following wash, cells were labeled with 250 μM 5-chloro-2′-deoxyuridine (CldU, Sigma, C6891) for 30 min. For nascent DNA stability assay, cells were labeled with IdU for 20 min followed by CldU labeling for 20 min. Cells were then treated with 10 mM hydroxyurea for 4 h. After washing, cells were harvested and suspended at 10^3^/μL in concentration. Cell suspension (2.5 μL) was dropped onto one end of the slide and incubated in 7.5 μL lysis buffer (50 mM EDTA; 0.5% SDS; 200 mM Tris-HCl [pH 7.5]) for 8 min at room temperature. The slide was then tilted to allow the drop flow slowly to form DNA fibers. After air drying, the slide was fixed with methanol/acetic acid (3:1) for 10 min and then treated with 2.5 M hydrochloric acid at 4 °C overnight. Immunofluorescent staining was performed using IdU and CIdU antibodies. DNA fibers were analyzed with Olympus FV1000 confocal microscope. The lengths (1 μm = 2.59 kb) of DNA fibers were measured with the Image J software. A total of 100 DNA fibers were examined for each sample, and each experiment was independently repeated twice.

### Immunoblotting

Immunoblotting was performed as previously described [17]. Cells were lysed with RIPA lysis buffer (Beyotime, P0013B) for 20 min in ice and centrifuged at 10,000*g* at 4 °C for 10 min. Supernatants were collected and mixed with 5×SDS-PAGE loading buffer (250 mM Tris-Hcl [pH 6.8], 10% SDS, 0.5% BPB, 50% glycerinum, 5% β-mercaptoethanol) and heated to 100 °C for 10 min. After electrophoresis, protein samples were transferred to PVDF membrane, which was blocked with blocking reagent (Roche, 11096176001) for 60 min followed by incubation with primary antibodies and second antibodies, respectively. Images were captured using a Protein SimpleFluorChem system. Each experiment was carried out in triplicate, and a representative blot is shown unless otherwise stated.

### Immunofluorescent staining

Cells are cultured on coverslips until approximately 70%–80% confluence. After fixing with 4% (w/v) paraformaldehyde for 15 min at room temperature, cells were permeabilized for 5 min with 0.25% TritonX-100 in PBS and blocked for 60 min with 1% bovine serum albumin. Cells were then incubated with primary and secondary antibodies. Images were captured using Olympus FV1000 confocal microscope.

### Laser micro-irradiation

Laser micro-irradiation was performed as described [78]. Cells were cultured on coverslips until approximately 70%–80% confluence and were micro-irradiated with a 405-nm pulse laser for 10 s using Olympus FV1000 confocal microscope. Cells were then cultured for another 2, 4, and 6 h, respectively, followed by fixation and immunostaining.

### Immunoprecipitation

hESCs (1 × 10^7^ cells) were harvested and lysed in 100 μL RIPA lysis buffer (Beyotime, P0013D) for 1 h on ice. After centrifuge at 10,000*g* at 4 °C for 10 min, the supernatants were collected and immunoprecipitation was carried out using Protein G Dynabeads (Thermo Fisher Scientific, 10004D) according to the manufacturers’ protocol. After immunoprecipitation, proteins were fractionated by SDS-PAGE gel followed by immunoblotting analysis.

### Neutral comet assay

Neutral comet assay was performed as described [36]. Briefly, glass slides were dipped into melted 0.8% agarose for 5 s and air dried. Single hESCs were resuspended in ice-cold PBS (Ca^2+^ and Mg^2+^ free) in a concentration of 1 × 10^6^ cells/mL. Cells (10 μL) were added into 70 μL 0.8% melted low-melting-point (LMP, Sangon Biotech, A600015-0025) agarose kept at 37 °C. The cell–agarose suspension was immediately pipetted and evenly spread on the prepared slides, covered by coverslip. Slides were then kept at 4 °C for 10 min and immersed in neutral lysis solution (2.5 M NaCl, 100 mM Na_2_EDTA, 10 mM Tris, 1% N-lauroylsarcosine, 1% TritonX-100 [pH 9.5]) for 60 min at room temperature without coverslip. Slides were washed and incubated in cold neutral electrophoresis buffer (300 mM sodium acetate, 100 mM Tris [pH 8.3]) for 30 min. Electrophoresis was carried out at 1 V/cm, 80 mA for 30 min. After electrophoresis, the slides were washed, fixed with anhydrous ethanol, and air dried. DNA was stained with 1 μg/mL DAPI (Invitrogen, D1306) for 30 min at room temperature. After washing with water three times, images were immediately analyzed using a Leica digital camera. Comets were analyzed by Komet 7 comet assay software (Andor Technology). A total of 100 cells were counted per slide, and each experiment was independently repeated twice.

### Sequencing of *KHDC3L gene*

DNA fragment covering the entire gene region of *KHDC3L* was amplified from genomic DNA of blood samples by forward primer 5′-TCTTCTGATTAGTGATGCGGA-3′ and reverse primer 5′-CCTTGAGTAGAACGACAGCG-3′ (PCR product: 3,650 bp). Three sequencing primers were used for sequencing the exonic region: 5′-CGCACGTCCAGGGTATGT-3′ for exon 1, 5′-CTCTTCTTCCAGGCTCAGG-3′ for exon 2, and 5′-CAGAGCCAGTCAGGGGTTA-3′ for exon 3 (including the deletions). The mutation rate of the deletion in patients was compared with that of controls by using Fisher’s exact test.

The heterozygous mutations of KHDC3L were confirmed by PCR amplification of a fragment in two patients with the following primers: forward primer: 5′-GATCCAGAAGGCCAAATTGAA-3′ and reverse primer: 5′-GATGCCTTCATAATCTAGTAAC-3′.

### shRNA knockdown of *KHDC3L* expression

*KHDC3L* knockdown was performed by using shRNA. pTRIPZ lentiviral tet-on inducible shRNAmir system was used to construct the shRNAmir expression vector according to the manufacture’s instruction (Open Biosystems). The 97mer oligonucleotide for the *KHDC3L* shRNAmir was 5′-TGCTGTTGACAGTGAGCGCACCCTTTCTGTTGCATGGTTGTAGTGAAGCCACAGATGTACAACCATGCAACAGAAAGGGTATGCCTACTGCCTCGGA-3′.

The pTRIPZ vector containing *KHDC3L* shRNAmir was cotransfected into the 293T cells with the packaging plasmids (psPAX2 and PMD2G) to package lentivirus. hESCs were transfected with lentivirus to establish the stable cell lines. hESCs were cultured with 0.5–2 μg/mL doxycycline for 48 h, followed by immunoblotting analysis of KHDC3L protein level.

### Quantification of the percentages of γH2AX-, active CASPASE-3-, or TUNEL-positive cells

We followed the method in previous study to quantify the percentages of γH2AX^+^, active CASPASE-3^+^, or TUNEL^+^ cells [79]. Images were visualized using a laser scanning confocal microscope (Olympus, FV1000) and captured with a 20× objective. Nuclei were segmented on the basis of DAPI staining. At least five noncontinuous sections from each teratoma were included, and at least two microscopic fields with more than 5,000 cells were calculated on each section.

### Statistical analysis

Data were analyzed by GraphPad Prism 5 (GraphPad Software, La Jolla, CA, United States). All data were presented as the mean ± SEM unless otherwise defined. The Student *t* test (two-tailed) was performed for comparisons.

## Supporting information

S1 Fig Establishment of hESC lines with deficit KHDC3L. (A) mRNA expressions of *KHDC3L* in human embryos at E5 through E7 (left panel) and in cynomolgus monkey epiblast cells from E7 through E17 (right panel). Expression data were downloaded from published database (accession numbers for the data used in this study: GSE74767 and E-MTAB-3929). (B) Scheme of CRISPR/Cas9 strategy to disrupt *KHDC3L* expression. (C) Sanger sequencing validated the disruption of the *KHDC3L* gene. (D) Immunoblotting confirmed the absence of KHDC3L protein expression in *KHDC3L*^−/−^ hESCs. (E) Immunoblotting showed the successful complementation of WT KHDC3L, Δ11, and Δ23 mutant proteins in *KHDC3L*^−/−^ hESCs. Underlying numerical values in A can be found in S1 Data. Δ11, p.E150_V160del; Δ23, p.E150_V172del; E, embryonic day; hESC, human embryonic stem cell; KHDC3L, KH domain containing 3 like; WT, wild-type.
(TIF)

S2 Fig Phenotypes of hESCs with knockout or mutation of KHDC3L. Compared to hESCs expressing WT KHDC3L (WT, WT-R), hESCs without KHDC3L (*KHDC3L*^−/−^) or with mutant KHDC3L (Δ11-R, Δ23-R) had normal morphology (A), pluripotency marker expression (B), cell proliferation rate (*n* = 3) (C), and cell phase distribution (*n* = 3) (D, E, F). Student two-tailed *t* test was performed for statistical analysis. Scale bars, 100 μm. Underlying numerical values in (C), (D), (E), and (F) can be found in S1 Data. Δ11, p.E150_V160del; Δ23, p.E150_V172del; hESC, human embryonic stem cell; KHDC3L, KH domain containing 3 like; WT, wild-type.
(TIF)

S3 Fig In vitro differentiation of hESCs through EB formation. Quantitative real-time PCR showed the continuous decrease in mRNA expressions of *NANOG* and *POU5F1* along with the EB differentiation. At day 10 of differentiation, all hESCs had undergone complete differentiation (*n* = 3). Underlying numerical values can be found in S1 Data. EB, embryoid body; hESC, human embryonic stem cell; *NANOG*, Nanog homeobox; *POU5F1*, POU class 5 homeobox 1.
(TIF)

S4 Fig KHDC3L is not involved in DNA replication stress response in hESCs. (A) DNA fiber assay revealed that the fork restart rates were comparable among hESCs with proficient (WT, WT-R, and T156D-R) or deficient KHDC3L (*KHDC3L*^−/−^, Δ11-R, Δ23-R, and T156A-R). Fork restart was examined at 30 min and 90 min following HU removal (*n* = 200 from two independent experiments). (B) hESCs with deficient KHDC3L (*KHDC3L*^−/−^, Δ11-R, Δ23-R, and T156A-R) had similar length of nascent DNA (CIdU labeled) when compared to cells with proficient KHDC3L (WT, WT-R, and T156D-R) under the normal (upper panel) or HU treatment condition (*n* = 200 from two independent experiments). (C) The ATR-CHK1 signaling was efficiently activated in hESCs with deficient KHDC3L (*KHDC3L*^−/−^, Δ11-R, and Δ23-R) when compared to cells with proficient KHDC3L (WT and WT-R). (D) KHDC3L was tagged with Flag. Cells were incubated with BrdU for 5 min to label the nascent DNA. Coimmunostaining revealed that KHDC3L did not localize on replication forks. Student two-tailed *t* test was performed for statistical analysis. Scale bars, 10 μm. Underlying numerical values in (A) and (B) can be found in S1 Data. Δ11, p.E150_V160del; Δ23, p.E150_V172del; ATR, Ataxia-telangiectasia and Rad3-related protein; BrdU, 5-bromo-2′-deoxyuridine; CHK1, checkpoint kinase 1; CldU, 5-chloro-2′-deoxyuridine; hESC, human embryonic stem cell; HU, hydroxyurea; KHDC3L, KH domain containing 3 like; WT, wild type.
(TIF)

S5 Fig KHDC3L deficiency impairs HR repair and PARP1 activation. (A) hESCs were subject to laser micro-irradiation to induce DNA DSBs. The kinetics of DSB repair was monitored by the percentages of γH2AX^+^ cells at different time points of recovery. WT hESCs showed efficient DSB repair, whereas *KHDC3L*^−/−^ or Δ11-R cells had compromised DSB repair (*n* = 50 in one replicate, total three independent replicates). (B) Compared to WT hESCs, hESCs without functional KHDC3L (*KHDC3L*^−/−^ or Δ11-R) were more sensitive to etoposide-induced DNA DSBs. (C) hESCs were subjected to laser micro-irradiation to induce DNA DSBs. The percentages of cells capable of performing HR repair (RAD51^+^γH2AX^+^ cells) were evaluated at different time points of recovery. The HR repair was compromised in hESCs without functional KHDC3L (*KHDC3L*^−/−^ or Δ11-R) (*n* = 50 in one replicate, total three independent replicates). (D) Apoptosis inhibitor z-DEVD-fmk successfully suppressed apoptosis and PARP1 cleavage. However, it did not affect the levels of PAR and γH2AX. (E) Suppression of apoptosis by two inhibitors did not affect DNA damage repair as assessed by neutral comet assay. (F) Suppression of apoptosis by two inhibitors did not affect HR-mediated DNA damage repair. Student two-tailed *t* test was performed for statistical analysis. Data are represented as mean ± SEM. *p < 0.05, **p < 0.01, ***p < 0.001. Underlying numerical values in (A), (C), (E), and (F) can be found in S1 Data. Δ11, p.E150_V160del; Δ23, p.E150_V172del; DSB, double-strand break; hESC, human embryonic stem cell; HR, homologous recombination; KHDC3L, KH domain containing 3 like; PAR, poly(ADP-ribose); PARP, PAR polymerase; WT, wild type; z-DEVD-fmk, Z-DEVD fluoromethylketone.
(TIF)

S6 Fig Inhibition of PARP1 did not affect HR repair. (A) hESCs with proficient KHDC3L (WT, WT-R) activated ATM-CHK2 signaling in response to Etop treatment, whereas hESCs with deficient KHDC3L (*KHDC3L*^−/−^, Δ11-R, and Δ23-R) failed to sustain the ATM-CHK2 signaling. (B-D) PARP1 activation was successfully inhibited by different PARP1 inhibitors AG14361 (B), talazoparib (C), and benzamide (D), respectively. At 2 h of recovery, recruitment of RAD51 to DSB sites (RAD51^+^γH2AX^+^ cells) was not influenced by PARP1 inhibition, indicating that HR repair does not rely on PARP1 activity (*n* = 50 in one replicate, total three independent replicates). Student two-tailed *t* test was performed for statistical analysis. Data are represented as mean ± SEM. Underlying numerical values in (B), (C), and (D) can be found in S1 Data. Δ11, p.E150_V160del; Δ23, p.E150_V172del; ATM, Ataxia-telangiectasia mutated; CHK2, checkpoint kinase 2; Etop, etoposide; hESC, human embryonic stem cell; HR, homologous recombination; KHDC3L, KH domain containing 3 like; PAR, poly(ADP-ribose); PARP1, PAR polymerase 1; RAD51, RAS associated with diabetes protein 51; WT, wild type.
(TIF)

S7 Fig Establishment of Δ11^−/−^ and Δ23^+/−^ hESC lines. (A) Sanger sequencing validated the deletion of 11 amino acids in two alleles (Δ11^−/−^) and the deletion of 23 amino acids in one allele (Δ23^+/−^). (B) Immunoblotting validated the precise deletion mutations in hESCs. Note that Δ23^+/−^ hESCs expressed similar amounts of WT and Δ23 mutant proteins. (C) KHDC3L knockdown by Dox-inducible shRNA. (D) Expression of WT KHDC3L, Δ11, and Δ23 mutant KHDC3L in WT hESCs. Underlying numerical values in (C) can be found in S1 Data. Δ11, p.E150_V160del; Δ23, p.E150_V172del; Dox, doxycycline; hESC, human embryonic stem cell; KHDC3L, KH domain containing 3 like; shRNA, short hairpin RNA; WT, wild-type.
(TIF)

S8 Fig Phosphorylation of T156 and T145 regulates the functions of KHDC3L. (A) Immunoblotting confirmed the establishment of hESC lines complemented with WT KHDC3L, T145A, T156A, T156D, and T145A/T156A mutant proteins, respectively. (B) hESCs were treated with 10 μM Etop. The ATM-CHK2 signaling was efficiently activated in WT and T156D-R cells but was similarly compromised in hESCs with deficient KHDC3L (T156A-R and Δ11-R). (C) The Δ11, T145A, or T156A mutation compromised ATM-CHK2 signaling to a similar extent, whereas T145A/T156A double mutation as well as KHDC3L knockout caused a more severe defect in ATM-CHK2 signaling. Δ11, p.E150_V160del; Δ23, p.E150_V172del; ATM, Ataxia-telangiectasia mutated; CHK2, checkpoint kinase 2; Etop, etoposide; hESC, human embryonic stem cell; KHDC3L, KH domain containing 3 like; WT, wild-type.
(TIF)

S1 Table Primers for PCR cloning and quantitative real-time PCR. (XLSX)

S2 Table Antibody information. (XLSX)

S1 Data (XLSX)

## Abbreviations

aa: amino acid
ATM: Ataxia-telangiectasia mutated
Atr: Ataxia-telangiectasia and Rad3-related protein
Brca2: breast cancer 2
cGAS: Cyclic GMP-AMP (cGAMP)
CHK1: checkpoint kinase 1
CHK2: checkpoint kinase 2
CMDB: Chinese Millionome Database
DSB: double-strand break
E: embryonic day
EB: embryoid body
ECAT1: ES cell-associated transcript 1
ESC: embryonic stem cell
hESC: human ESC
HR: homologous recombination
IB: immunoblot
IgG: immunoglobulin G
*KHDC3L*: KH domain containing 3 like
Mcm2-7: Minichromosome maintenance protein 2–7
NANOG: Nanog homeobox
NHEJ: nonhomologous end-joining
NLRP7: NLR family pyrin domain containing 7
NOD/SCID: nonobese diabetic/severe combined immunodeficiency
ns: not significant
PAR: poly(ADP-ribose)
PARP: PAR polymerase
POU5F1: POU class 5 homeobox 1
Rad51: RAS associated with diabetes protein 51
RPL: recurrent pregnancy loss
shRNA: short hairpin RNA
SSB: single-strand break
STING: synthase stimulator of interferon genes
TUNEL: TdT-mediated dUTP Nick-End Labeling
WT: wild type
z-DEVD-fmk: Z-DEVD fluoromethylketone
Δ11: p.E150_V160del
Δ23: p.E150_V172del
