# Supplementary figures and images for: KHDC3L mutation causes recurrent pregnancy loss by inducing genomic instability of human early embryonic cells
Source: PLoS Biol. 2019 Oct 14;17(10):e3000468. doi: 10.1371/journal.pbio.3000468 (PMC6812846; doi:10.1371/journal.pbio.3000468)

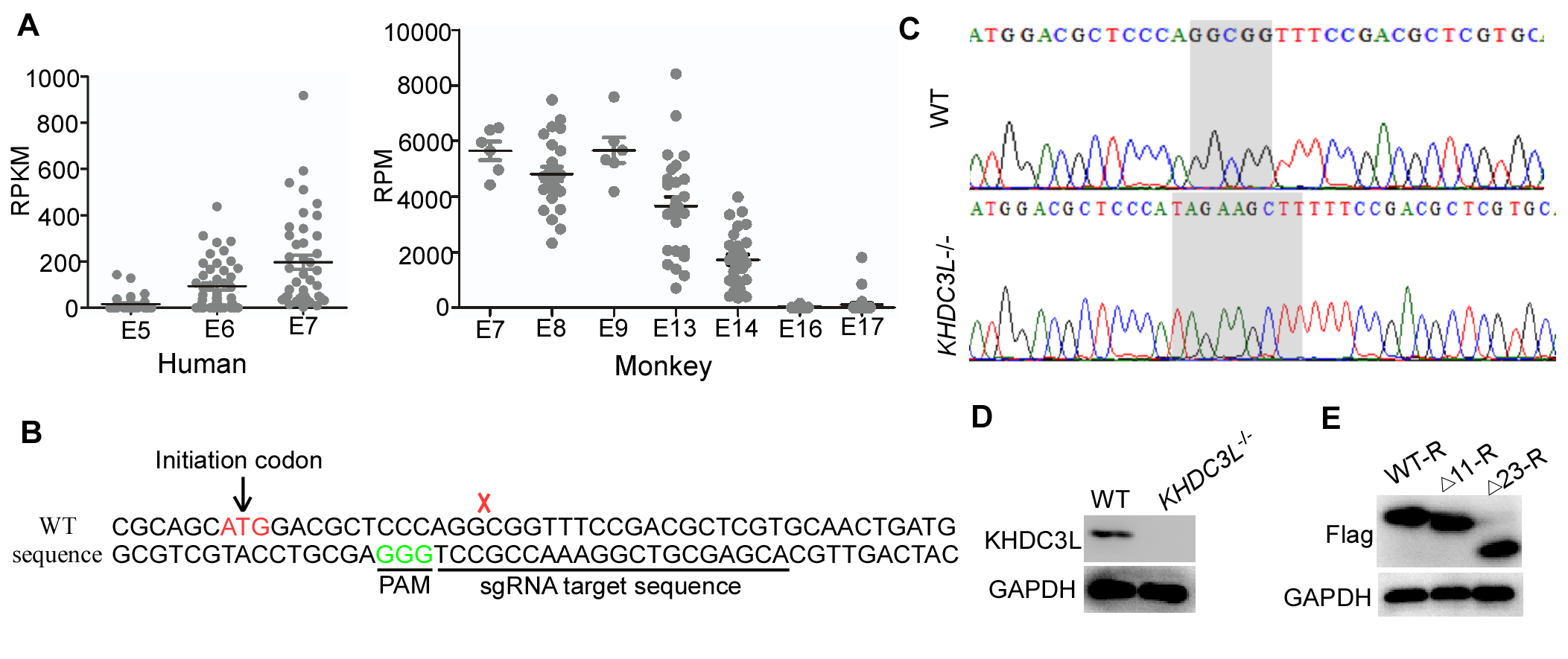

Supplement: S1 Fig — (A) mRNA expressions of KHDC3L in human embryos at E5 through E7 (left panel) and in cynomolgus monkey epiblast cells from E7 through E17 (right panel). Expression data were downloaded from published database (accession numbers for the data used in this study: GSE74767 and E-MTAB-3929). (B) Scheme of CRISPR/Cas9 strategy to disrupt KHDC3L expression. (C) Sanger sequencing validated the disruption of the KHDC3L gene. (D) Immunoblotting confirmed the absence of KHDC3L protein expression in KHDC3L−/− hESCs. (E) Immunoblotting showed the successful complementation of WT KHDC3L, Δ11, and Δ23 mutant proteins in KHDC3L−/− hESCs. Underlying numerical values in A can be found in S1 Data. Δ11, p.E150_V160del; Δ23, p.E150_V172del; E, embryonic day; hESC, human embryonic stem cell; KHDC3L, KH domain containing 3 like; WT, wild-type. (TIF) [file pbio.3000468.s001.tif]

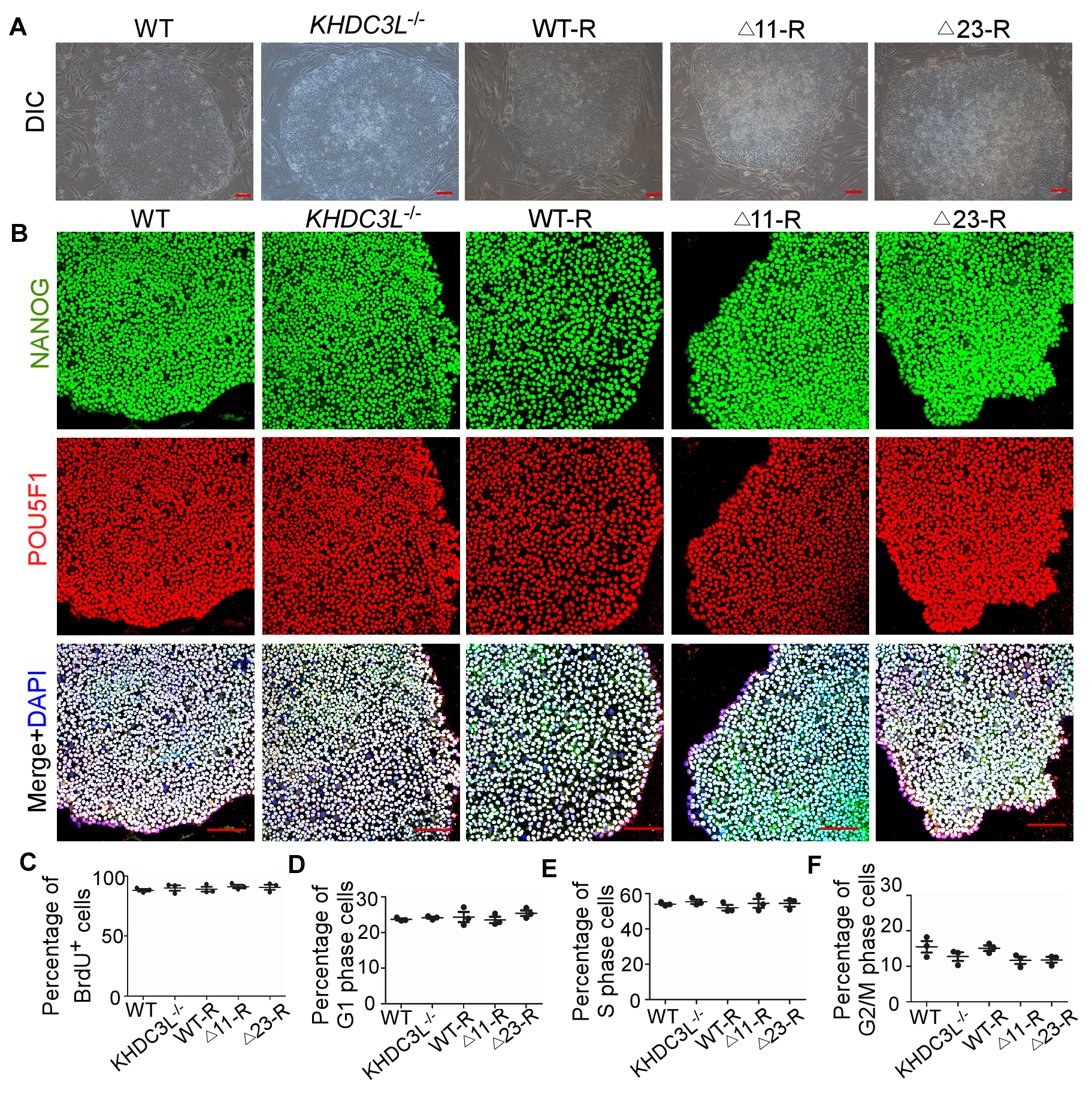

Supplement: S2 Fig — Compared to hESCs expressing WT KHDC3L (WT, WT-R), hESCs without KHDC3L (KHDC3L−/−) or with mutant KHDC3L (Δ11-R, Δ23-R) had normal morphology (A), pluripotency marker expression (B), cell proliferation rate (n = 3) (C), and cell phase distribution (n = 3) (D, E, F). Student two-tailed t test was performed for statistical analysis. Scale bars, 100 μm. Underlying numerical values in (C), (D), (E), and (F) can be found in S1 Data. Δ11, p.E150_V160del; Δ23, p.E150_V172del; hESC, human embryonic stem cell; KHDC3L, KH domain containing 3 like; WT, wild-type. (TIF) [file pbio.3000468.s002.tif]

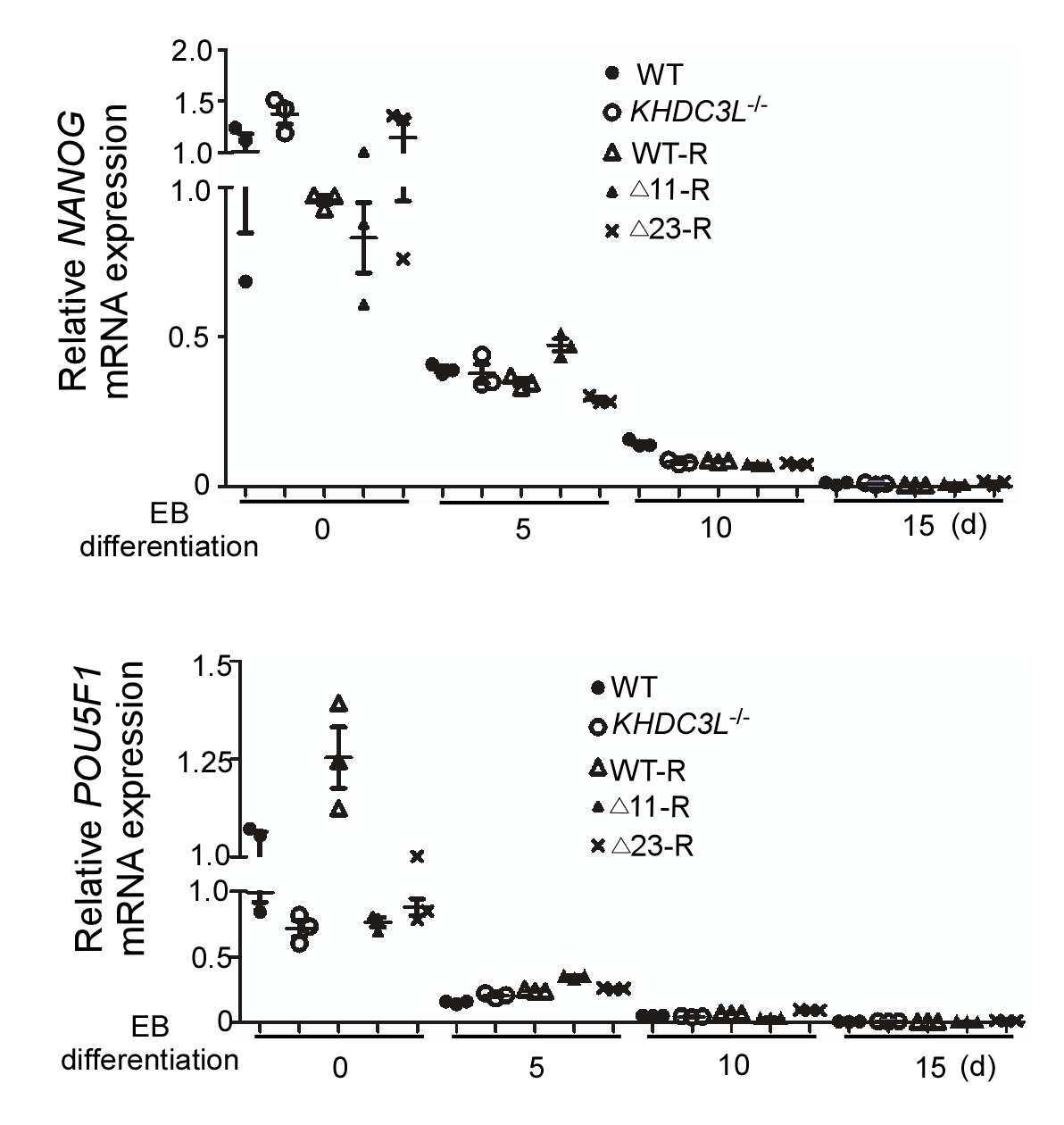

Supplement: S3 Fig — Quantitative real-time PCR showed the continuous decrease in mRNA expressions of NANOG and POU5F1 along with the EB differentiation. At day 10 of differentiation, all hESCs had undergone complete differentiation (n = 3). Underlying numerical values can be found in S1 Data. EB, embryoid body; hESC, human embryonic stem cell; NANOG, Nanog homeobox; POU5F1, POU class 5 homeobox 1. (TIF) [file pbio.3000468.s003.tif]

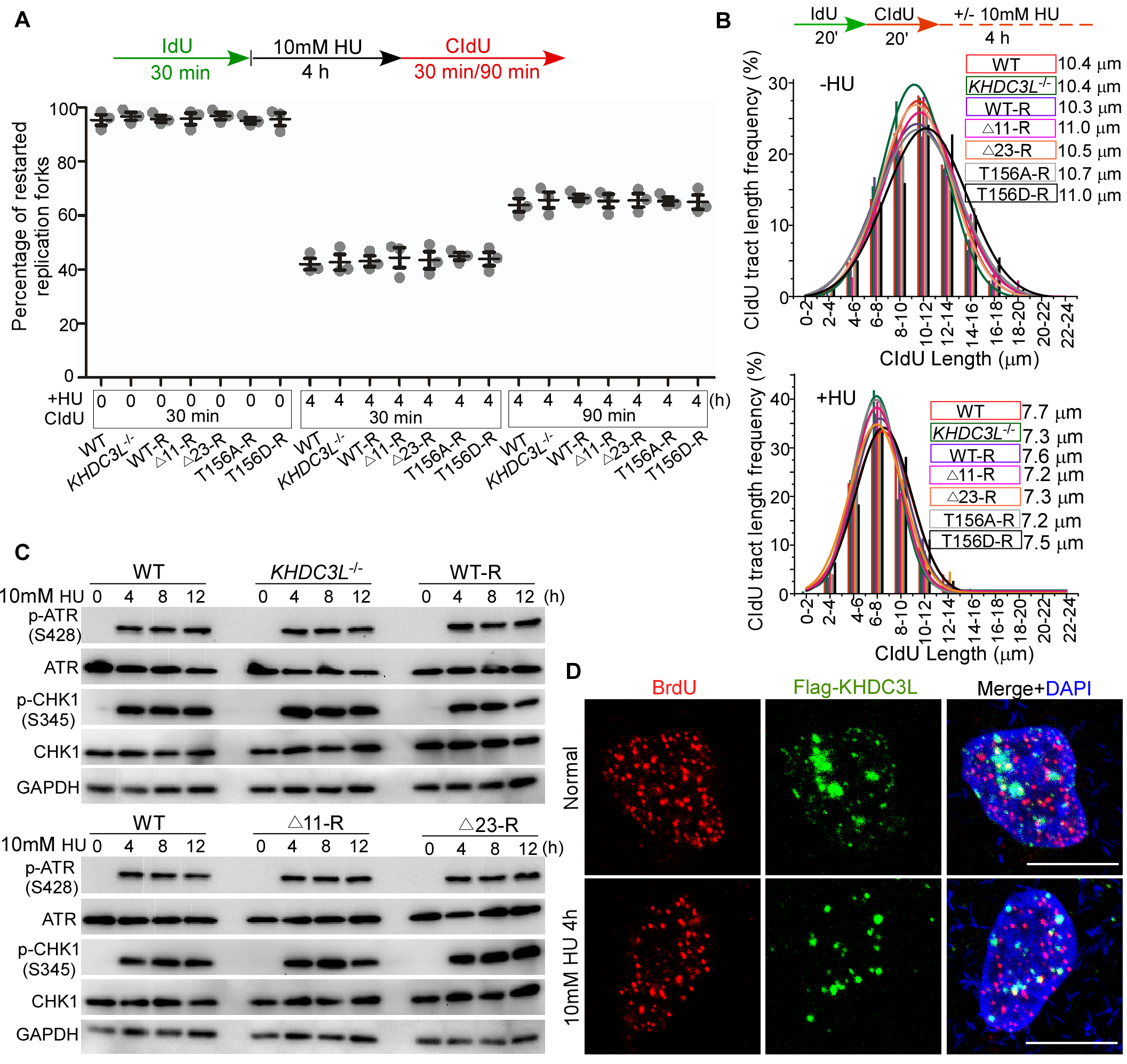

Supplement: S4 Fig — (A) DNA fiber assay revealed that the fork restart rates were comparable among hESCs with proficient (WT, WT-R, and T156D-R) or deficient KHDC3L (KHDC3L−/−, Δ11-R, Δ23-R, and T156A-R). Fork restart was examined at 30 min and 90 min following HU removal (n = 200 from two independent experiments). (B) hESCs with deficient KHDC3L (KHDC3L−/−, Δ11-R, Δ23-R, and T156A-R) had similar length of nascent DNA (CIdU labeled) when compared to cells with proficient KHDC3L (WT, WT-R, and T156D-R) under the normal (upper panel) or HU treatment condition (n = 200 from two independent experiments). (C) The ATR-CHK1 signaling was efficiently activated in hESCs with deficient KHDC3L (KHDC3L−/−, Δ11-R, and Δ23-R) when compared to cells with proficient KHDC3L (WT and WT-R). (D) KHDC3L was tagged with Flag. Cells were incubated with BrdU for 5 min to label the nascent DNA. Coimmunostaining revealed that KHDC3L did not localize on replication forks. Student two-tailed t test was performed for statistical analysis. Scale bars, 10 μm. Underlying numerical values in (A) and (B) can be found in S1 Data. Δ11, p.E150_V160del; Δ23, p.E150_V172del; ATR, Ataxia-telangiectasia and Rad3-related protein; BrdU, 5-bromo-2′-deoxyuridine; CHK1, checkpoint kinase 1; CldU, 5-chloro-2′-deoxyuridine; hESC, human embryonic stem cell; HU, hydroxyurea; KHDC3L, KH domain containing 3 like; WT, wild type. (TIF) [file pbio.3000468.s004.tif]

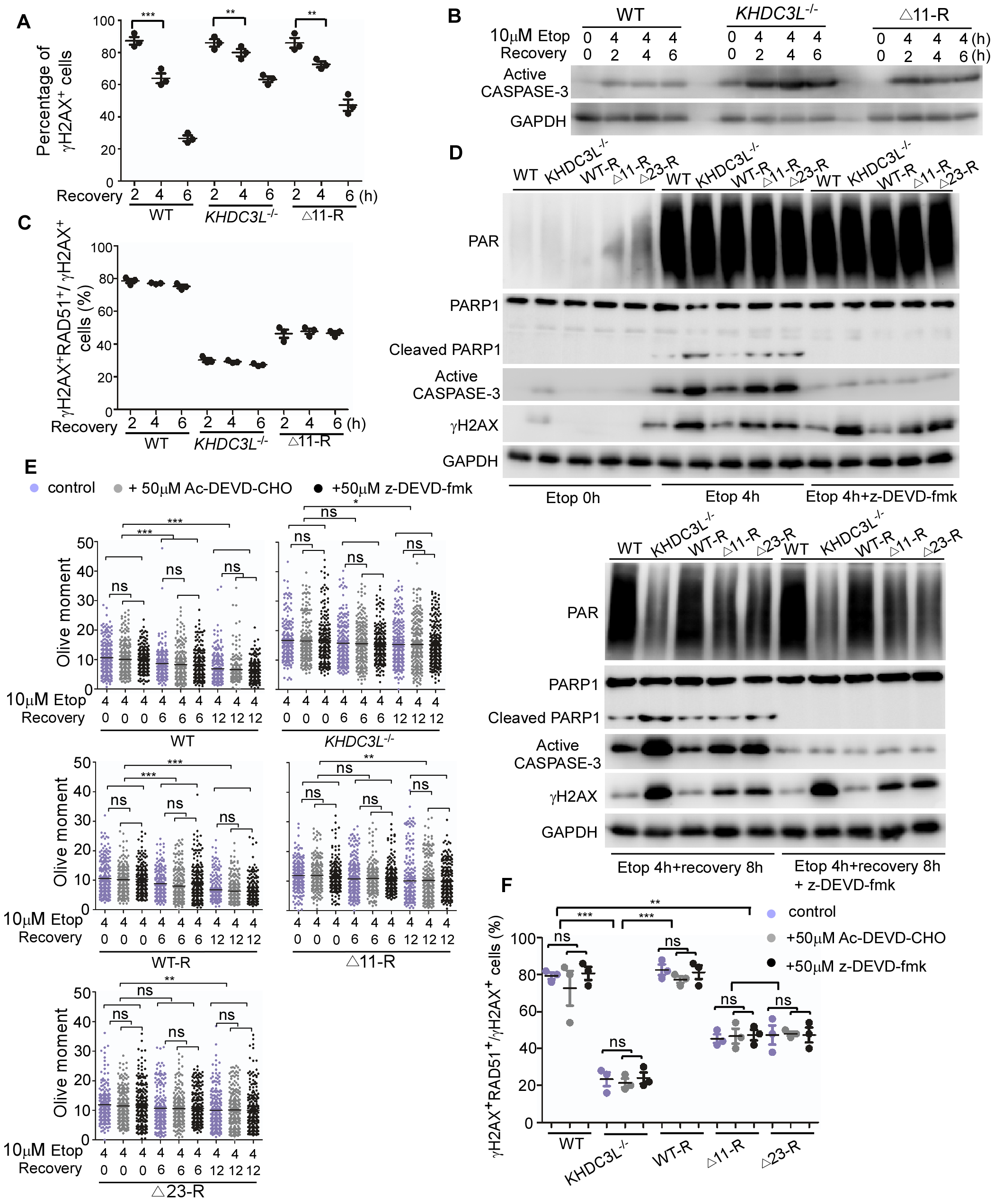

Supplement: S5 Fig — (A) hESCs were subject to laser micro-irradiation to induce DNA DSBs. The kinetics of DSB repair was monitored by the percentages of γH2AX+ cells at different time points of recovery. WT hESCs showed efficient DSB repair, whereas KHDC3L−/− or Δ11-R cells had compromised DSB repair (n = 50 in one replicate, total three independent replicates). (B) Compared to WT hESCs, hESCs without functional KHDC3L (KHDC3L−/− or Δ11-R) were more sensitive to etoposide-induced DNA DSBs. (C) hESCs were subjected to laser micro-irradiation to induce DNA DSBs. The percentages of cells capable of performing HR repair (RAD51+γH2AX+ cells) were evaluated at different time points of recovery. The HR repair was compromised in hESCs without functional KHDC3L (KHDC3L−/− or Δ11-R) (n = 50 in one replicate, total three independent replicates). (D) Apoptosis inhibitor z-DEVD-fmk successfully suppressed apoptosis and PARP1 cleavage. However, it did not affect the levels of PAR and γH2AX. (E) Suppression of apoptosis by two inhibitors did not affect DNA damage repair as assessed by neutral comet assay. (F) Suppression of apoptosis by two inhibitors did not affect HR-mediated DNA damage repair. Student two-tailed t test was performed for statistical analysis. Data are represented as mean ± SEM. *p < 0.05, **p < 0.01, ***p < 0.001. Underlying numerical values in (A), (C), (E), and (F) can be found in S1 Data. Δ11, p.E150_V160del; Δ23, p.E150_V172del; DSB, double-strand break; hESC, human embryonic stem cell; HR, homologous recombination; KHDC3L, KH domain containing 3 like; PAR, poly(ADP-ribose); PARP, PAR polymerase; WT, wild type; z-DEVD-fmk, Z-DEVD fluoromethylketone. (TIF) [file pbio.3000468.s005.tif]

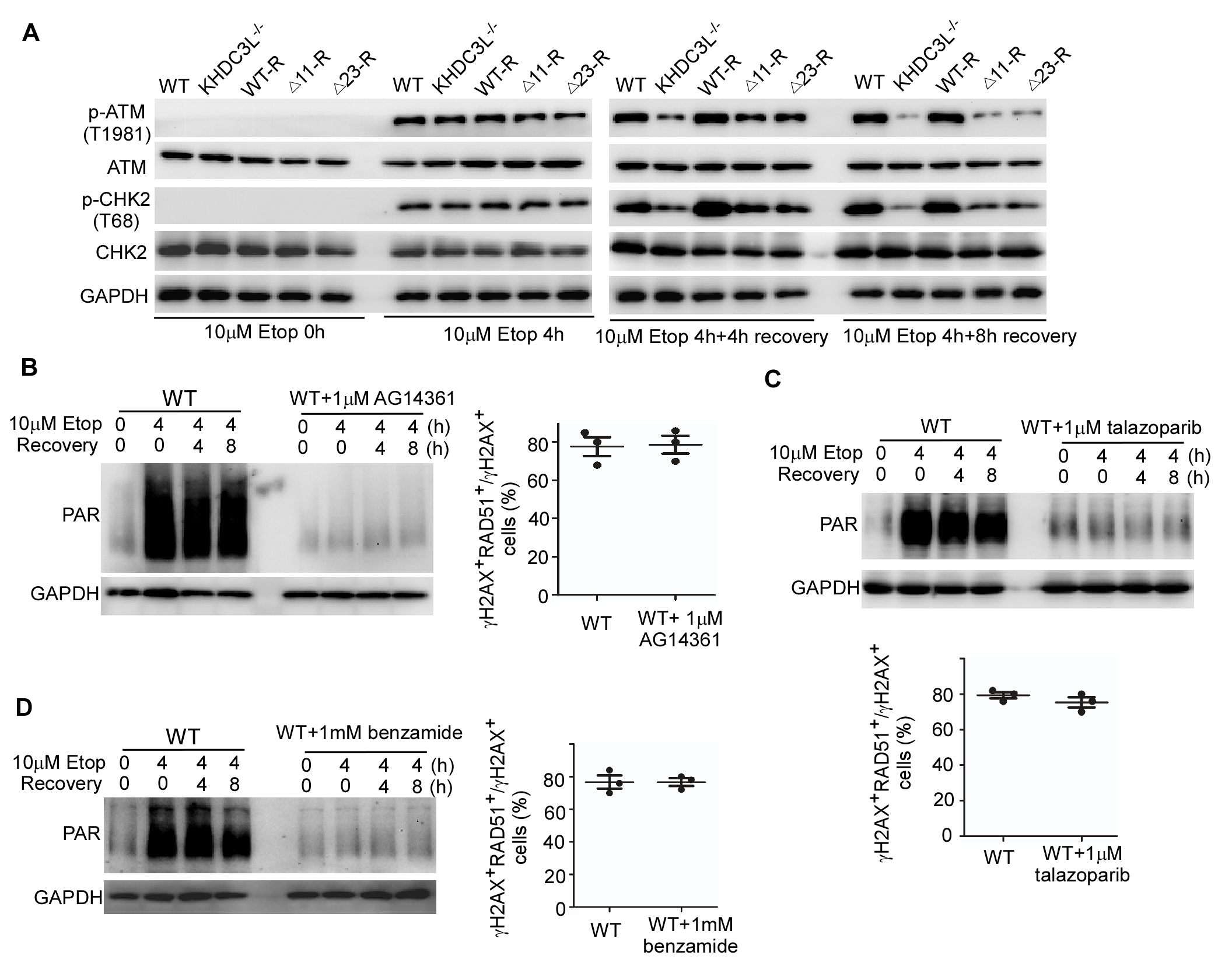

Supplement: S6 Fig — (A) hESCs with proficient KHDC3L (WT, WT-R) activated ATM-CHK2 signaling in response to Etop treatment, whereas hESCs with deficient KHDC3L (KHDC3L−/−, Δ11-R, and Δ23-R) failed to sustain the ATM-CHK2 signaling. (B-D) PARP1 activation was successfully inhibited by different PARP1 inhibitors AG14361 (B), talazoparib (C), and benzamide (D), respectively. At 2 h of recovery, recruitment of RAD51 to DSB sites (RAD51+γH2AX+ cells) was not influenced by PARP1 inhibition, indicating that HR repair does not rely on PARP1 activity (n = 50 in one replicate, total three independent replicates). Student two-tailed t test was performed for statistical analysis. Data are represented as mean ± SEM. Underlying numerical values in (B), (C), and (D) can be found in S1 Data. Δ11, p.E150_V160del; Δ23, p.E150_V172del; ATM, Ataxia-telangiectasia mutated; CHK2, checkpoint kinase 2; Etop, etoposide; hESC, human embryonic stem cell; HR, homologous recombination; KHDC3L, KH domain containing 3 like; PAR, poly(ADP-ribose); PARP1, PAR polymerase 1; RAD51, RAS associated with diabetes protein 51; WT, wild type. (TIF) [file pbio.3000468.s006.tif]

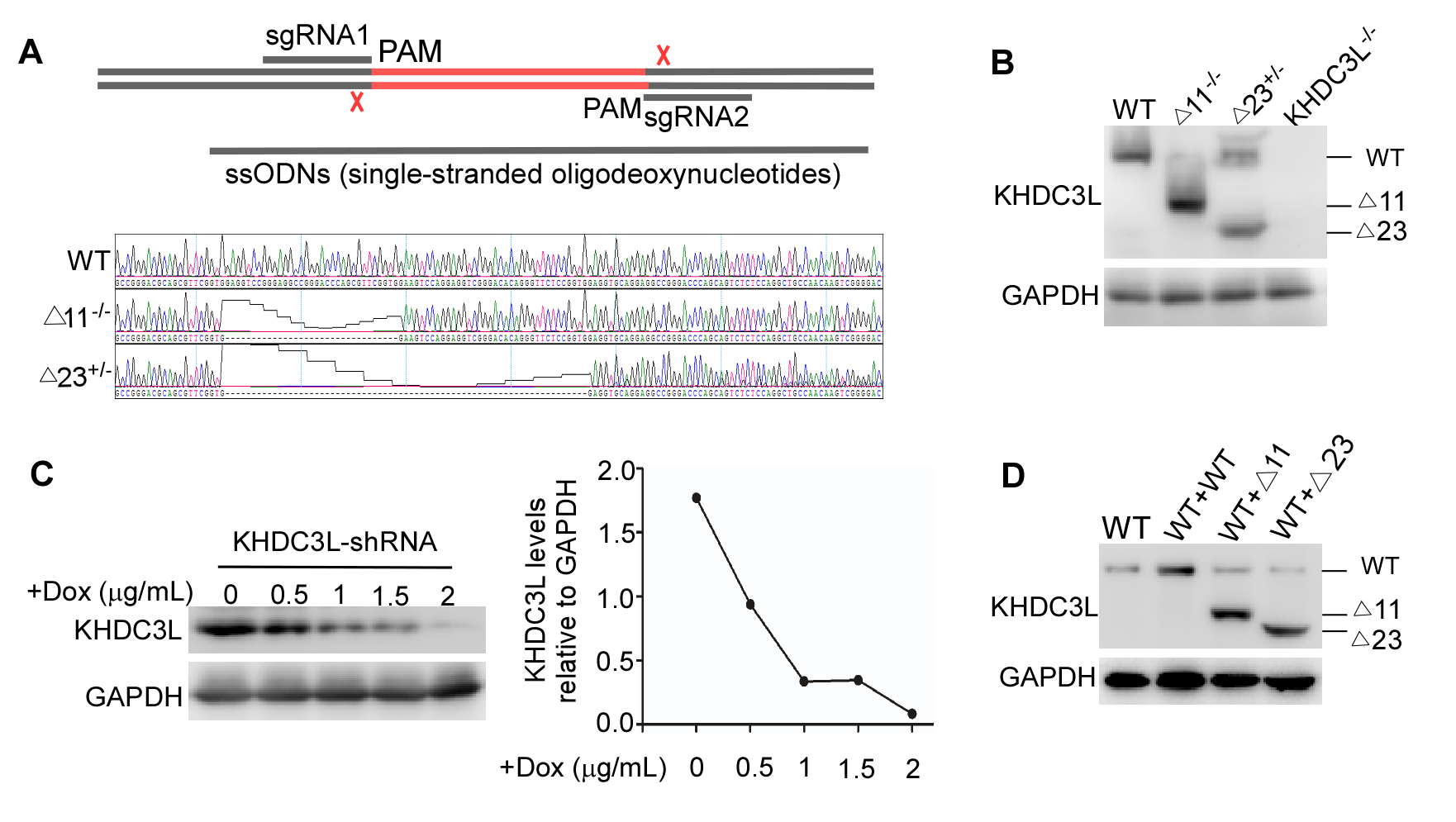

Supplement: S7 Fig — (A) Sanger sequencing validated the deletion of 11 amino acids in two alleles (Δ11−/−) and the deletion of 23 amino acids in one allele (Δ23+/−). (B) Immunoblotting validated the precise deletion mutations in hESCs. Note that Δ23+/− hESCs expressed similar amounts of WT and Δ23 mutant proteins. (C) KHDC3L knockdown by Dox-inducible shRNA. (D) Expression of WT KHDC3L, Δ11, and Δ23 mutant KHDC3L in WT hESCs. Underlying numerical values in (C) can be found in S1 Data. Δ11, p.E150_V160del; Δ23, p.E150_V172del; Dox, doxycycline; hESC, human embryonic stem cell; KHDC3L, KH domain containing 3 like; shRNA, short hairpin RNA; WT, wild-type. (TIF) [file pbio.3000468.s007.tif]

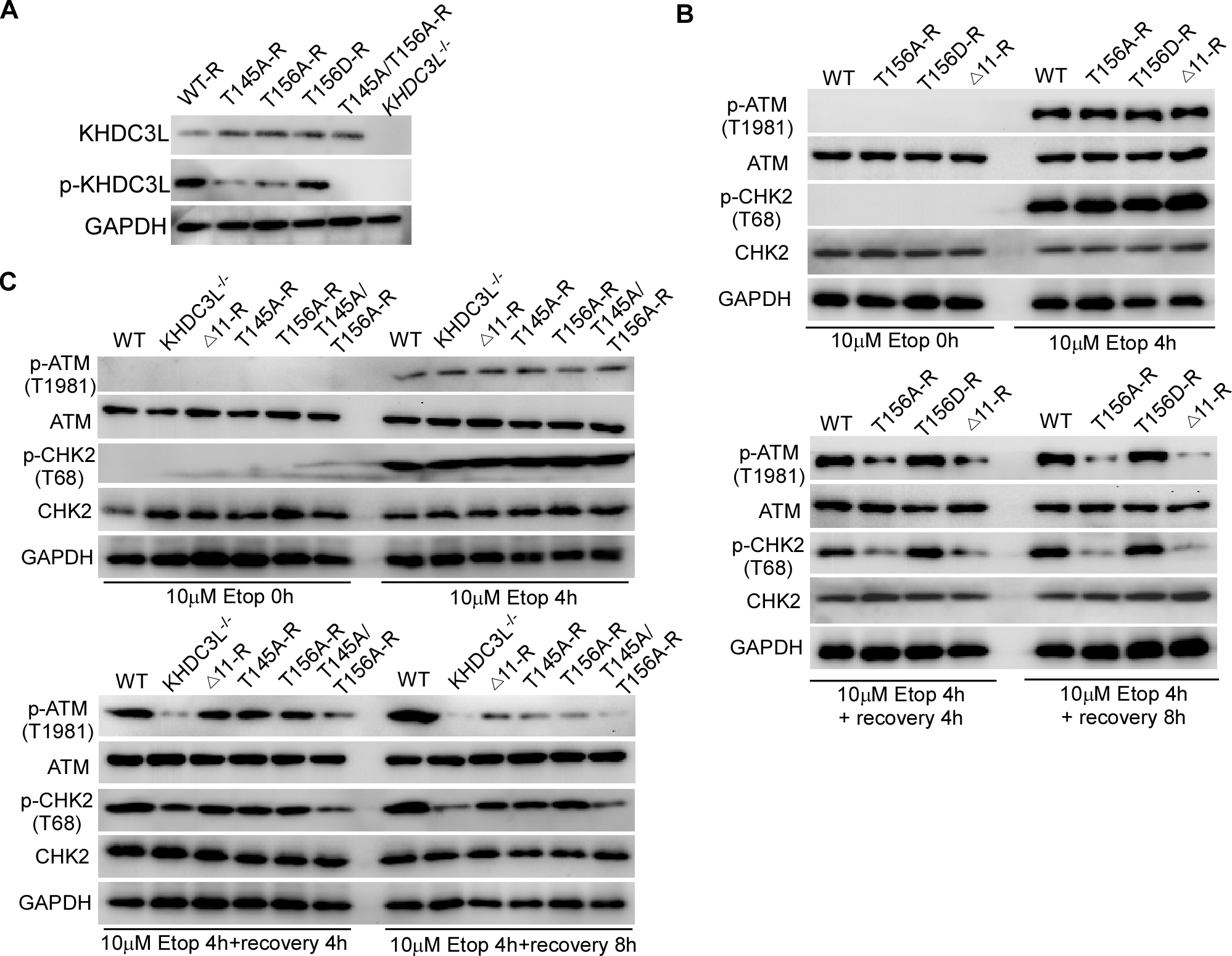

Supplement: S8 Fig — (A) Immunoblotting confirmed the establishment of hESC lines complemented with WT KHDC3L, T145A, T156A, T156D, and T145A/T156A mutant proteins, respectively. (B) hESCs were treated with 10 μM Etop. The ATM-CHK2 signaling was efficiently activated in WT and T156D-R cells but was similarly compromised in hESCs with deficient KHDC3L (T156A-R and Δ11-R). (C) The Δ11, T145A, or T156A mutation compromised ATM-CHK2 signaling to a similar extent, whereas T145A/T156A double mutation as well as KHDC3L knockout caused a more severe defect in ATM-CHK2 signaling. Δ11, p.E150_V160del; Δ23, p.E150_V172del; ATM, Ataxia-telangiectasia mutated; CHK2, checkpoint kinase 2; Etop, etoposide; hESC, human embryonic stem cell; KHDC3L, KH domain containing 3 like; WT, wild-type. (TIF) [file pbio.3000468.s008.tif]
